# Drivers of benthic metacommunity structure along tropical estuaries

**DOI:** 10.1038/s41598-020-58631-1

**Published:** 2020-02-03

**Authors:** Andreia Teixeira Alves, Danielle Katharine Petsch, Francisco Barros

**Affiliations:** 1 Laboratório de Ecologia Bentônica, Programa de Pós-Graduação em Ecologia: Teoria, Aplicação e Valores, Instituto de Biologia & CIENAM, Universidade Federal da Bahia, Rua Barão de Geremoabo s/n., Campus Ondina, CEP 40170-115 Salvador, BA Brazil; 2 Núcleo de Pesquisas em Limnologia, Ictiologia e Aquicultura (Nupelia), Programa de Pós-Graduação em Ecologia de Ambientes Aquáticos Continentais (PEA), Universidade Estadual de Maringá, Av. Colombo 5790, CEP 87020–900 Maringá, PR Brazil

**Keywords:** Community ecology, Community ecology

## Abstract

Community structure of many systems changes across space in many different ways (e.g., gradual, random or clumpiness). Accessing patterns of species spatial variation in ecosystems characterized by strong environmental gradients, such as estuaries, is essential to provide information on how species respond to them and for identification of potential underlying mechanisms. We investigated how environmental filters (i.e., strong environmental gradients that can include or exclude species in local communities), spatial predictors (i.e., geographical distance between communities) and temporal variations (e.g., different sampling periods) influence benthic macroinfaunal metacommunity structure along salinity gradients in tropical estuaries. We expected environmental filters to explain the highest proportion of total variation due to strong salinity and sediment gradients, and the main structure indicating species displaying individualistic response that yield a continuum of gradually changing composition (i.e., Gleasonian structure). First we identified benthic community structures in three estuaries at Todos os Santos Bay in Bahia, Brazil. Then we used variation partitioning to quantify the influences of environmental, spatial and temporal predictors on the structures identified. More frequently, the benthic metacommunity fitted a quasi-nested pattern with total variation explained by the shared influence of environmental and spatial predictors, probably because of ecological gradients (i.e., salinity decreases from sea to river). Estuarine benthic assemblages were quasi-nested likely for two reasons: first, nested subsets are common in communities subjected to disturbances such as one of our estuarine systems; second, because most of the estuarine species were of marine origin, and consequently sites closer to the sea would be richer while those more distant from the sea would be poorer subsets.

## Introduction

Understanding how community structure of many systems changes across space and how mechanisms, driven mostly by dispersal and environmental filters, determine species distribution patterns in local communities is a central question in community ecology^1–3^. Testing how community assembly mechanisms determine species distribution has also become important in metacommunity ecology, an offshoot of community ecology, which has emerged to describe processes occurring at local and regional scales^1,4^. A metacommunity can be defined as a set of local communities potentially, but not necessarily, linked by the dispersal of multiple, likely interacting, species^5,6^. Assessing processes that affect metacommunity composition particularly in ecosystems characterized by strong environmental gradients is important to provide useful information on species responses to environmental changes across ecological gradients.

To understand patterns of spatial variation in species composition, two different and complementary metacommunity approaches have been proposed^7^: one focusing on patterns^7,8^ and another focusing on mechanisms^1,9^. The pattern-based approach evaluates the characteristics of species distributions along environmental gradients (i.e., random, checkerboard, nested subsets, evenly-spaced, Gleasonian, or Clementsian patterns)^7,8^ (Table 1). The mechanistic approach considers the roles of niche (i.e., environmental filters and biotic interactions) and dispersal-related processes in determining such metacommunity structures. Both approaches have provided insights into the different processes that structure communities across different ecosystems^10–13^, but have not been applied along well-defined ecological gradients.

**Table 1:** Six idealized structures to identify species distribution among sites. Patterns represent idealized characteristics hypothesized as a result of ecological processes. References indicates early description of these patterns, Description explains each pattern, and Processes is a potentially important ecological or biogeographical cause.

Pattern	Reference	Description	Processes
Checkerboard	Diamond 1975	Combinations of mutually exclusive species that occur independently of other pairs along the gradient.	Biotic process may prevent coexistence of particular sets of species that interact antagonistically.
Nested subsets	Patterson and Atmar 1986	Species-poor communities are subsets of species-richer communities.	Species-specific characteristics, such as dispersal ability and tolerance to abiotic conditions.
Clementsian	Clements 1916	Groups of species show similar responses to environmental gradients, which replace each other as a group, and can be classified into distinctive community types.	Biotic process may prevent coexistence of particular sets of species that interact antagonistically.
Gleasonian	Gleason 1926	Communities are structured along some gradient, but species display individualistic responses that yield a continuum of gradually changing composition.	Idiosyncratic responses to abiotic factors, with coexistence resulting from change similarities in requirements or tolerance.
Evenly spaced gradients	Tilman 1982	Species are distributed more uniformly than expected by chance.	Strong interspecific competition.
Random	Simberloff 1983	There are no gradients or other patterns in species distribution among sites.	Indicator of stochastic processes.

**Figure 1:**
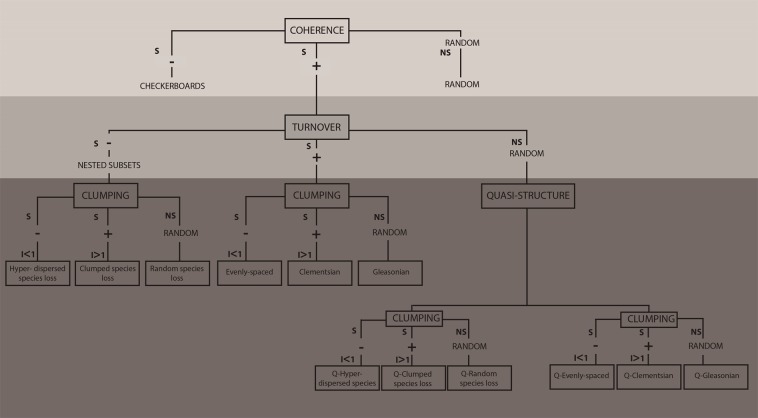
Schematic representation used to examine how the EMS (i.e., coherence, turnover and boundary clumping) results in six main metacommunity structures (i.e., checkerboards, random, nested, evenly spaced, Gleasonian and Clementsian) and quasi-structures. S = significant; NS = non-significant; “ + ” = positive; “−” = negative; “I” = Morisita’s index value. Modified from Presley *et al*.^7^ and Brasil *et al*.^65^.

The framework devised by Leibold and Mikkelson^8^ to identify patterns of metacommunity structure (later expanded^7^) is based on evaluating three metrics – coherence, turnover and boundary clumping (known as Elements of Metacommunity Structure, herein called EMS) – calculated from a presence–absence matrix^8,14^. Coherence, the first element of the hierarchical EMS framework, is related to the level at which species respond to the same environmental gradient, while turnover relates to the way species composition changes across communities, and boundary clumping measures the level of distinctiveness of blocks of species. Following coherence, turnover and boundary clumping, it is possible to identify species’ distribution patterns among sites (Fig. 1). Accordingly, EMS analyzes multiple models simultaneously, comparing them against each other to assess which one best fits a particular metacommunity pattern along a single major ordination axis (i.e., a latent environmental gradient) in the data^7,8,10,15^. Among the idealized structures for identifying species distributions among sites, community structure can change across space in many different ways (e.g., gradual, random, clumpiness) (Table 1). However, EMS indicates but does not directly inform the processes underlying patterns, such as the role of environmental filtering or dispersal effects in metacommunity structuring^2,4^. Therefore, combining the EMS approach with variation partitioning techniques (environmental and spatial variation) is strongly recommended to assess the main drivers of observed metacommunity structure^3^. For instance, different functional groups of freshwater benthic invertebrate communities^12^ may display Clementsian or random patterns (identified by the pattern-based approach) likely due to different causes (identified by the mechanistic approach), respectively environmental heterogeneity and dispersal mode.

Strong environmental gradients, like salinity gradients in estuaries, may be important metacommunity structure drivers, generating non-random and ecologically meaningful patterns^3,10,16^. Such gradients can be understood as environmental filters that can include or exclude species, at different sites along the gradient, through species interactions with the physical and chemical characteristics of the environment^3,17^. Environmental filters may be more pronounced than dispersal limitation or interactions between species in environments with strong environmental gradients. Furthermore, spatial variation plays an important role on the arrangement of environmental gradients and consequently on communities’ final distribution, but there is still a lack of formal tests on strong gradients that include spatial information when analyzing metacommunity structure patterns^3,10,12,18^. Temporal variation, such as sampling periods, should also be taken into account in metacommunity studies, since communities are not static, but dynamic^16,19,20^, notably in estuaries^21,22^. In addition, the relative roles of different local and regional processes in determining community structure and metacommunity pattern remain unclear in estuaries.

Estuarine systems are characterized by their transitional position between marine and freshwater ecosystems, which can generate strong and well-defined gradients (i.e., salinity and sediment grain size)^23,24^. It is well known that benthic communities play important roles in estuaries and, considering that component species have sedentary or relatively low mobility habits, changes in environmental gradients are frequently detected in benthic organisms resulting in benthic community changes^23^. Hence, the environmental estuarine gradient along with the benthic community life mode represents an opportunity to explore metacommunity patterns. Also, knowing the type of structure and the drivers shaping metacommunities in estuaries is important for providing information on how species respond to salinity gradients and, consequently, on the underlying mechanisms responsible for the general functioning of these systems^21,26,27^. Thus, estuaries are highly productive systems and provide several goods and services as they are often used as feeding areas and nursery grounds by various species, and serve as natural pollution filters and storm buffers^21,24^. However, despite their high ecological and economic importance, estuaries experience a wide array of human impacts, such as increased urbanization and industrialization and altered connection to marine and freshwater systems due to shoreline development^25^. These influences can compromise their ecological integrity and consequently change the structure of benthic assemblages. Environmental condition changes in systems characterized by strong gradients such as estuaries may reflect alterations in species replacement^9,22^, and impacts over time might modify the organisms’ ranges along the salinity gradient^23^, highlighting the importance of knowing how such communities are structured and the main drivers of structure.

We used an empirical framework linking the EMS approach and variation partitioning (environmental, spatial and temporal variation)^3^ to understand the emergence of benthic macroinfaunal metacommunity structure along salinity gradients in tropical estuaries. Even though benthic species diversity patterns along tropical estuaries^21,22^ (decreasing from marine to freshwater zones) had challenged previous well-accepted paradigms (i.e., Remane, 1934^28^), metacommunity patterns of estuarine species are still unclear. This study offers insight into the debates on diversity patterns along estuaries through a metacommunity approach. We first expected a Gleasonian pattern (i.e., species displaying individualistic responses producing a continuum of gradually changing composition), since estuarine benthic fauna had been previously associated with ecocline^23^ and species replacement^22^ ideas. Thus, we predicted that the benthic metacommunity would be organized according to salinity preferences^21–23^ following a non-clumped association (i.e., Gleasonian distribution). Due to the strong salinity gradient, we expected environmental filters to be more important in explaining benthic community variation than were spatial and temporal predictors.

## Materials and Methods

### Study area

**Figure 2:**
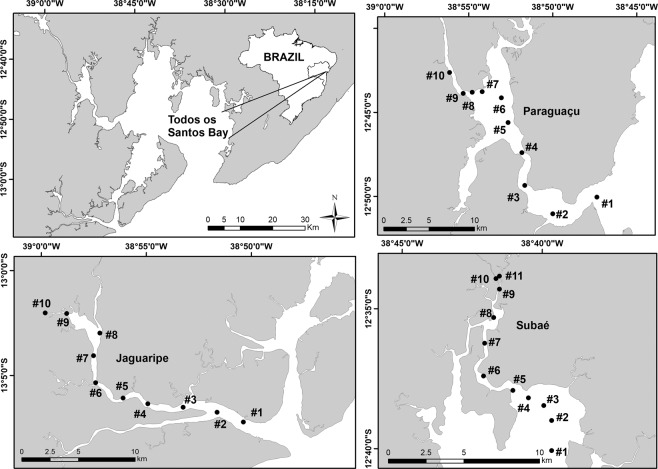
Map of the study area showing sampled stations (black dots) for each estuary (Subaé, Jaguaripe, and Paraguaçu) at Todos os Santos Bay, in Bahia, Brazil.

**Figure 3:**
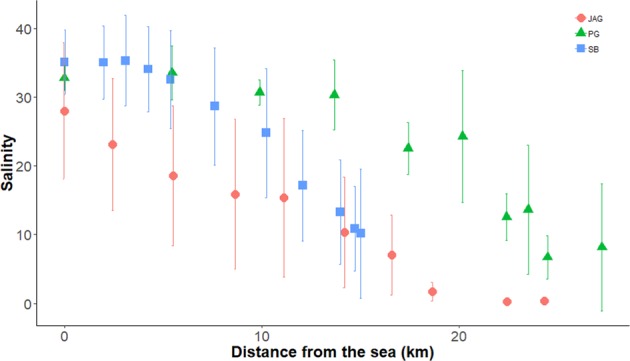
Salinity gradient from sea to freshwater in sampled sites for Jaguaripe (circles), Paraguaçu (triangles) and Subaé (squares) estuaries at Baía de Todos os Santos.

We conducted our study in the estuarine portion of the three main tributaries of Todos os Santos Bay located in Bahia state in Brazil: Paraguaçu (56,300 km^2^), Subaé (600 km^2^) and Jaguaripe (2,200 km^2^) Rivers^29^ (Fig. 2). Several anthropogenic activities, such as industrial effluents, untreated sewage, urbanization, agriculture, ports and mining activities, have decreased the environmental quality in some specific regions of our study area^30^. Since we were interested in analyzing the influence of environmental filters on the structure of metacommunities, each estuary studied encompassed the effect of a salinity gradient ranging from approximately 0.5 to 40 along 10 or 11 randomly chosen stations (Fig. 3). Each one of the 10 (Jaguaripe and Paraguaçu) or 11 stations (Subaé) along the salinity gradient had two randomly chosen sites. A total of 270 sites were sampled for all estuarine systems over time. In each estuary, we sampled in a gradient from the most seaward and generally deepest station (i.e., lower-numbered stations in Fig. 2) to the furthest inland and shallowest station (i.e., higher-numbered stations in Fig. 2).

The survey took place over time in different estuaries. The Subaé estuary was sampled in five periods: Jun-2004, Mar-2006, Dec-2009, Apr-2011, and Mar-2013. The Jaguaripe estuary was sampled in four periods: May-2006, Aug-2007, Jul-2010, and Aug-2014. Finally, the Paraguaçu estuary was sampled in four periods: May and Dec-2005, Jun-2011, and Aug-2014.

### Sample collection and processing

In the Paraguaçu estuary, the six replicates were collected at each station using a van Veen grab (0.05 m^2^, 3.2 L). In Subaé and Jaguaripe, at each station eight replicates were collected manually by divers, using corers (10 cm diameter, 0.008 m^2^, 1.2 L). Core sampling was not suitable in the Paraguaçu River due to the depth at some stations ( > 35 m), very strong water current and zero visibility. For both types of gear, sample infauna was collected from the water–sediment interface to a depth of 15 cm^31^. All macroinfaunal samples were sieved through a 0.5 mm mesh in the field, preserved in 70% alcohol and taken to the laboratory for further processing and identification. Most invertebrates were identified to family level; family has been shown to be a taxonomically sufficient descriptor of estuarine benthic invertebrates in habitats with strong gradients^32^ but still provide information about community identities and their temporal drift^33^. Also, family level was a good choice due to the scarcity of taxonomical studies of the local benthic invertebrates (with several undescribed species) and allowed comparison of the taxon distribution patterns observed in other regions^21^.

The environmental variables measured included salinity and sediment type (i.e., grain size). Salinity of the superficial water was measured at spring low ebb tides and recorded using a Hydrolab Data Sonde. One sediment sample was collected at each station for grain size analysis, using a 0.05 m^2^ van Veen grab for Paraguaçu River and a 0.008 m^2^ corer for Subaé and Jaguaripe. Sediment particle size was determined by standard techniques^34^. Salinity and each fraction of sediment grain size (i.e., pebble, gravel, very coarse sand, coarse sand, medium sand, fine sand, very fine sand and silt sand clay) were treated as environmental predictors.

### Sample adequacy

In order to evaluate whether the benthic macroinfaunal family of each estuary was representatively sampled and to avoid artifactual patterns because the probability of detection of species varied, we calculated the relationship between sampling effort and family richness for each estuary for the total sampling time. The *specaccum* function and the species accumulation method *random* were used in the vegan package^35^ in the R environment^36^. Sample-based rarefaction is preferable than individual-based rarefaction to account for natural levels of sample heterogeneity in the data^37^.

A total of 11,328 individuals of benthic invertebrates were sampled, mainly belonging to 144 taxa of Polychaeta, Mollusca and Crustacea. Polychaeta was the most abundant phylum, followed by Mollusca and Crustacea. In spite of differences in the sampling methods (i.e., sampling gear, total area sampled, number of sites and replicates) among estuaries, most of the systems showed a near stabilization of the relationship between number of stations and richness, allowing further analyses (Supplementary Fig. 1).

### Elements of metacommunity structure

We used incidence matrices (i.e., presence–absence) to estimate Elements of Metacommunity Structure (EMS). We followed the ‘range perspective’ in our analysis, which is defined by species range turnover and range boundary clumping^8^ as recommended^38^. These incidence matrices were subsequently subjected to a reciprocal averaging (also known as Correspondence Analysis, CA), an unconstrained ordination method, which positions sites having similar species composition close to each other and locating species having similar occurrence among the sites close to each other along the ordination axis^39^. Because we focused only on the first ordination axis, other ordination methods, such as detrended correspondence analysis, will give the same results^8^. When a single or at least a predominant gradient structures a community data set, the reciprocal averaging arranged matrix will display this gradient effectively.

*Coherence*, the first EMS metric, is based on calculating the number of embedded absences (i.e., interruptions in species distribution or in the composition of the sites) in the ordinated matrix and then comparing the empirical observed value of embedded absences (EmbAbs) to a null distribution created from simulated matrices with 1,000 iterations^7,8^. A large number of embedded absences (i.e., EmbAbs significantly larger than expected by chance) suggests negative coherence and leads to a checkerboard distribution of species; non-significant coherence refers to a random metacommunity type; and a small number of embedded absences (i.e., EmbAbs significantly lower than expected by chance) suggests positive coherence related to nestedness, evenly spaced, Gleasonian or Clementsian gradients^8^ (Fig. 1).

*Turnover* is evaluated if coherence is significant and positive (Fig. 1). It is measured by the number of times one species replaces another between two sites (i.e., number of replacements) in an ordinated matrix. To do this, the number of empirical replacements (turnover) was compared to the distribution of randomly generated values based on a null model distribution that randomly shifts entire ranges of species^8^. Significant negative turnover (i.e., replacement significantly lower than expected by chance) refers to nested subsets, while significant positive turnover (i.e., replacement significantly larger than expected by chance) refers to evenly spaced gradients (Gleasonian or Clementsian structures), requiring further analysis of boundary clumping to distinguish among them^8^. Furthermore, cases where coherence is significant and positive and turnover is non-significant can be regarded as quasi-structures, indicating that the effects of structuring mechanisms are weaker than in idealized structures^7^ (Fig. 1).

*Boundary clumping* is analysed using the Morisita’s Index^40^ and a chi-square test comparing observed and expected distributions of range boundary locations. Non-significant clumping, and values of Morisita’s index that are not different from 1, indicate randomly distributed species loss in nested subsets when turnover is negative or Gleasonian distribution when turnover is positive. Values significantly larger than 1 indicate clumped species loss in nested subsets when turnover is negative or Clementsian distribution when turnover is positive. Values significantly less than 1 indicate hyperdispersed species loss in nested subsets when turnover is negative and an evenly spaced metacommunity type when turnover is positive (Fig. 1).

The significance of coherence and turnover was tested separately using the fixed-proportional null model, where the species richness of each site was maintained (i.e., row sums were fixed), but species frequencies of occurrence (i.e., columns) were filled based on their marginal probabilities. Random matrices were produced by the *r1* method using the R package vegan^35^ for the fixed-proportional null model, which has a more desirable combination of Type I and Type II error properties^7^ and has been applied successfully^15,16,38,41,42^. All EMS analyses were done using the metacom package^43^ in the R environment (version 1.5.0)^36^.

### Nestedness

We performed ‘nestedness metric based on overlap and decreasing fill’ (NODF)^44^ to accurately identify nestedness along salinity gradients in estuarine benthic communities. There is some criticism about the EMS framework used to investigate idealized metacommunity patterns and especially whether the turnover test is adequate for detecting a nested pattern, as turnover and nestedness are not necessarily exclusive or opposite^38,45,46^. Schmera *et al*.^46^ showed that even though high turnover is frequently related to low nestedness, low turnover does not predict high nestedness. We performed NODF using the *oecosimu* function from the vegan package^35^ in the R environment (version 2.0-10)^36^.

### Spatial predictors

We used Principal Components of Neighbour Matrices (PCNM) to generate spatial variables from geographical coordinates (e.g., latitude and longitude) represented as a Euclidean distance matrix^47,48^. The PCNM technique represents the spatial configuration of sample points using principal coordinates of a truncated geographic distance matrix between sampling sites. We used the resulting PCNM eigenvectors associated with the positive eigenvalues as spatial components in a global test and in a forward selection prior to variation partitioning^47,49,50^. PCNM analyses were done using the function *pcnm* in the vegan package^35^ in the R environment (version 2.0-10)^36^.

### Environmental predictors

We converted environmental data to standardized Z-scores by subtracting each environmental variable from their mean and dividing by their standard deviation. The new standardized variables are thus dimensionless, with a mean of 0 and a standard deviation of 1^51^. In addition, we tested multicollinearity using a variance inflation factor (VIF)^52^. When the VIF values indicated a high level of collinearity, we removed the predictor with the highest VIF value. We then recalculated VIF and repeated this process until all VIFs were below a pre-selected threshold (VIF < 3)^52,53^. Standardized Z-score and VIF analyses were done using the functions *scale* and *vif.cca*, respectively, in the vegan package^35^ in the R environment (version 2.0-10)^36^.

### Variation partitioning

We used partial Redundancy Analysis (pRDA)^51^ to quantify the pure and shared contributions of environmental filters (i.e., salinity and each fraction of sediment grain size), spatial variables (i.e., variables created using PCNM) and time (i.e., sampling occasions) structuring the benthic metacommunity in the estuaries. RDA can be best understood as an extension of multiple regression that has multiple response variables (i.e., species) and a common matrix of predictors (i.e., environmental and spatial predictors)^54^. pRDA or variation partitioning^51^ may indicate the relative strength of association between each component and the metacommunity pattern of benthic macroinvertebrates. We expected environmental variables to be the main influencer of benthic metacommunity structure. In situations where environmental gradients determine most of the variation in the living community, the amount of variation in species data explained by environmental variables is fairly high^55^. We also included sampling period as a temporal predictor in variation partitioning because time may influence community structure, but we did not have specific expectations regarding temporal variation in benthic structures.

Prior to the pRDA, we Hellinger-transformed abundance matrices and report values based on adjusted R^2^ to provide unbiased estimates of explained variation and valid comparisons between sets of factors for explaining community structure^54^. Hellinger transformation consists of transforming the site-by-species data into relative values per site by dividing each value by the site sum, and then taking the square root of the resulting values^42^. It is suitable for community composition data in comparative analysis because it reduces the importance of high species abundance.

We first did a global RDA test to prevent the inflation of Type I error, and only if it was significant proceeded with forward selection using the double-stopping criterion: the usual alpha significance level (p < 0.05) and the adjusted coefficient of multiple determination (R^2^)^49^. Each eigenvector was counted as a single predictor since this approach is the most conservative in its penalization of degrees of freedom and adjusted R^2^ statistics^50^. We used forward selection to determine the environmental and spatial filters to be used in variation partitioning. Forward selection analyses for spatial and environmental predictors were done using the *ordiR2step* function in the vegan package^35^ in the R environment (version 2.0-10)^36^.

We carried out pRDA using the function *varpart* in the vegan package^35^ in the R environment (version 2.0-10)^36^. We report adjusted R^2^ and test the significance of the pure environmental, pure spatial and pure temporal components (P < 0.05). The total percentage of variation explained by the model (R^2^) is partitioned into unique and common contributions of sets of predictors^54^. It offers a way of dealing with the importance of spatial correlation when observations are not independent, the number of degrees of freedom in the sample is smaller than expected based on the number of observations used in the analysis, and Type I errors increase, leading to incorrect conclusions about the effect of the environment on community structure^56^. Statistical significance of RDA in global models was based on 999 permutations and assessed at a significance level of 0.05.

## Results

### Elements of metacommunity structure

The EMS analysis indicated five metacommunity patterns among the six idealized patterns^57–62^ and the quasi-structures^7^ (Table 2). We found that the Q-nested (n = 6) metacommunity type structure was the most common followed by nested (n = 3), Q-Clementsian (n = 2), Clementsian (n = 1), and Q-Gleasonian (n = 1) (Supplementary Fig. 2). As expected, for all estuaries the first step of EMS analysis (Fig. 1), indicated that metacommunity structure was positively coherent (*P* < 0.001). That is, EmbAbs was significantly lower than expected by chance (Table 2) likely due to the salinity gradient (Supplementary Fig. 2). The second EMS step (Fig. 1) revealed that turnover was not significant (*P* > 0.05) in most cases (9 out of 13), displaying quasi-structures^7^ and predominantly negative turnover. That is, replacement was lower than expected by chance) (Table 2) (Supplementary Fig. 2). Even though spatial turnover among sites was more often linked to environmental gradients^49^, these results indicated that benthic macroinfaunal metacommunities did not always follow a species replacement structure. Finally, the boundary clumping third step (Fig. 1), showed that Morisita’s index was higher (*P < *0.005) than 1 for most (11) of the cases, indicating positive clumping structures or clumped species loss for the Q-nested and nested structures (Supplementary Fig. 2).

**Table 2:** The Elements of Metacommunity Structure results for each estuary (Subaé, Jaguaripe and Paraguaçu). These results were based on the fixed-proportional (r1) null model. Interpretations followed Leibold & Mikkelson^8^ and Presley *et al*.^7^. Abbreviations: embABS = embedded absences; Coh Z = Z-value of coherence; Tur Z = Z-value of turnover; Q = Quasi. Significant p-values are indicated by bold font.

Estuary Month Year	Coherence					Turnover					Boundary clumping			
	embAbs	Coh Z	*P*	Sim mean	Sim sd	Turnover	Tur Z	*P*	Sim mean	Sim sd	Index	*P*	df	Interpretation
Subaé 03 2013	95	6.52	**< 0.001**	179	13	921	−0.37	0.716	875	126	1.04	0.267	8	Q-Gleasonian
Subaé 04 2011	94	3.14	**< 0.001**	131	12	537	1.10	0.271	658	111	1.41	**0.008**	8	Q-nested*
Subaé 12 2009	101	7.58	**< 0.001**	280	24	886	6.01	**< 0.001**	2228	2229	4.29	**0.001**	8	Nested*
Subaé 03 2006	57	7.26	**< 0.001**	196	19	962	2.94	**< 0.001**	1296	117	1.20	0.107	8	Nested#
Subaé 06 2004	36	8.54	**< 0.001**	130	11	911	1.55	0.123	1095	119	4.00	**0.001**	7	Q-nested*
Jaguaripe 08 2014	85	1.06	**< 0.001**	210	12	2103	−0.27	0.787	2024	290	2.08	**0.001**	7	Q-Clementsian
Jaguaripe 07 2010	104	6.07	**< 0.001**	193	15	1150	1.25	0.204	1374	179	1.73	**0.009**	7	Q-nested*
Jaguaripe 08 2007	98	6.79	**< 0.001**	184	13	1058	0.36	0.721	1113	156	1.74	**0.008**	7	Q-nested*
Jaguaripe 05 2006	35	5.71	**< 0.001**	749	7	339	−1.10	0.270	287	47	1.74	**0.002**	6	Q-Clementsian
Paraguaçu 08 2014	263	1.01	**< 0.001**	392	13	3895	0.99	0.320	4270	378	1.36	**0.001**	6	Q-nested*
Paraguaçu 06 2011	130	1.18	**< 0.001**	288	14	3481	0.37	0.715	3651	469	1.95	**0.003**	7	Q-nested*
Paraguaçu 12 2005	91	1.13	**< 0.001**	253	15	2195	258	< **0.001**	2852	255	1.99	**0.008**	7	Nested#
Paraguaçu 05 2005	89	1.01	**< 0.001**	229	14	1886	−2.02	**< 0.05**	1527	177	1.35	**0.001**	7	Clementsian

*Nested clumped speceis loss (sensu Presley *et al*.^7^), Q = Quasi. #*Nested* random speceis loss (sensu Presley *et al*.^7^), Q = Quasi.

### Nestedness

**Figure 4:**
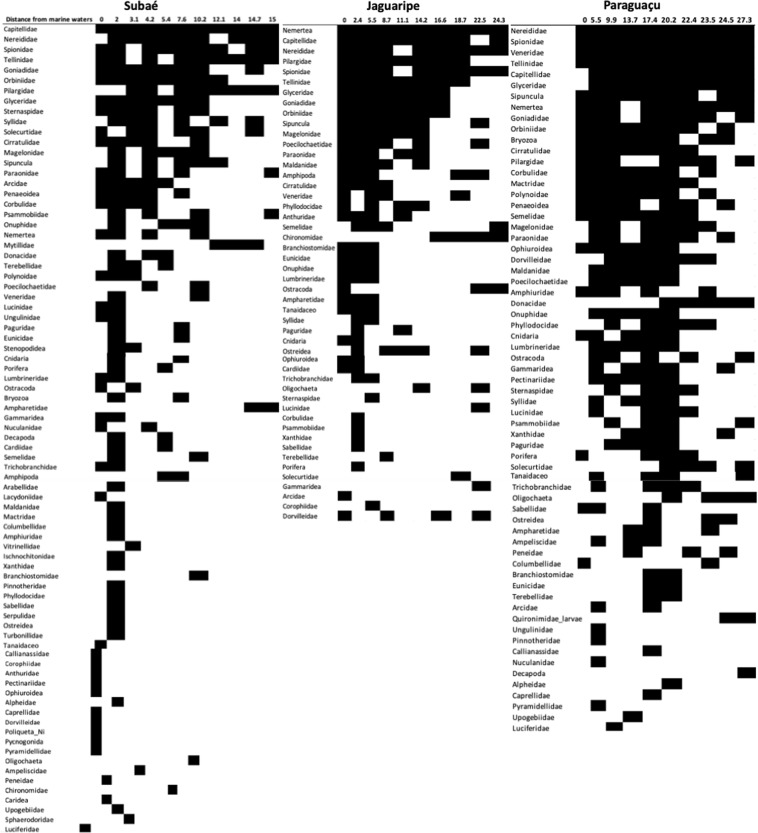
Taxa vs. sites, incidence matrices along the salinity gradient at the sampled stations for Jaguaripe, Paraguaçu and Subaé estuaries after ordination according to occurrence resulting from the overall NODF analysis. Black squares indicates the presence of a taxon, while white squares indicates the absence of a taxon along the salinity gradient indicated as distance to marine waters (km).

NODF results suggested that in Subaé and Paraguaçu systems, benthic macroinfaunal metacommunity followed an intermediate nested distribution for while the Jaguaripe system had a highly nested structure (Supplementary Table 1). Taxa vs. sites occurrence resulting from the overall NODF analysis based on incidence matrices along salinity gradients for Todos os Santos Bay estuarine systems showed that most of the taxa found follow Q-nested and nested species composition (Fig. 4).

### Variation partitioning

The RDA used in the spatial global model was significant for all estuaries (Subaé: adjusted R^2^ = 0.28, *P < *0.001; Jaguaripe: adjusted R^2^ = 0.48, *P < *0.001; Paraguaçu: adjusted R^2^ = 0.26, *P < *0.001), so forward selection was carried out to select spatial variables among PCNM eigenvectors associated with the positive eigenvalues before variation partitioning (Table 3).

**Table 3:** Environmental and spatial factors selected through forward selection, after checking multicollinearity using variance inflation factor (VIF) for each variable, during the sampled periods in the three main tributaries (Subaé, Jaguaripe and Paraguaçu) at Todos os Santos Bay.

Estuarine system	Spatial selected variables	Environmental selected variables	VIF
Subaé	PCNM 1, 3, 2, 5	Salinity	1.72
		Medium sand	1.81
		Fine sand	1.29
		Pebble	1.39
Jaguaripe	PCNM 1, 2	Coarse sand	1.92
		Salinity	1.70
		Very fine sand	1.47
		Medium sand	1.50
Paraguaçu	PCNM 1, 3, 2	Fine sand	1.78
		Salinity	2.21

The multicollinearity test for environmental predictors for all systems did not show a high VIF value for salinity even before dropping the predictor with the highest VIF value, indicating that there was no problem of multicollinearity among salinity and other predictors. However, sediment predictors showed a high VIF value and the predictor with highest value (VIF < 3) was removed for each system (Subaé: coarse sand, granular gravel, and silt/clay; Jaguaripe: fine sand, very coarse sand, and silt/clay; Paraguaçu: coarse sand, granular gravel and silt/clay). The remaining variables had a VIF value smaller than the threshold (Supplementary Table 2). The environmental global model was significant (Subaé: adjusted R^2^ = 0.24, *P = *0.001; Jaguaripe: adjusted R^2^ = 0.38, *P = *0.001; Paraguaçu: adjusted R^2^ = 0.25, *P = *0.001), so forward selection of environmental variables was also carried out to select environmental filters after the removal of collinear explanatory variables (Table 3).

**Figure 5:**
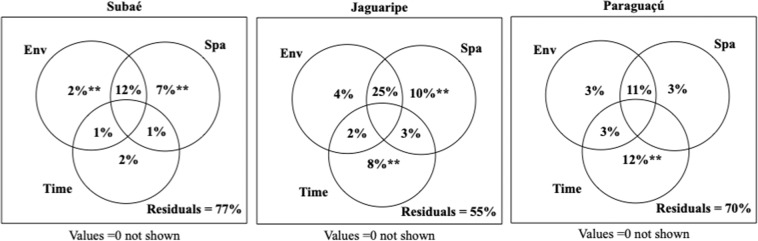
Explained proportion of variance partitioning for each estuarine system (Subaé, Jaguaripe and Paraguaçu): the effects related to environmental filters (*Env*), those related to spatial patterns (*Spa*), those resulting from temporal patterns (*Time*), shared influence of environmental filters and spatial descriptors (*Env* + *Spa*), environmental filters and temporal patterns (*Env* + *Time*), and spatial descriptors and temporal patterns (*Spa* + *Time*). *Numbers* indicate the explained proportion of variation partitioning for each estuarine system; values = 0 are not shown. **P < 0.01 (Supplementary Table 3).

The shared influence of environmental and spatial predictors explained a high proportion of benthic metacommunity structure (Fig. 5). For example, in the Subaé estuary, the shared influence of environmental and spatial predictors explained 12% of the variance, and spatial factors alone explained 7% (Fig. 5). Similarly, shared environmental and spatial predictors explained 25% and spatial predictors explained 10% in the Jaguaripe estuary. (Fig. 5). Finally, in the Paraguaçu estuary, temporal components explained 12% of metacommunity structure followed by 11% explained by shared environmental and spatial components (Fig. 5). Purely environmental, purely spatial and purely temporal predictors were significant (P < 0.01) influences in all three estuaries (Supplementary Table 3).

## Discussion

We used EMS combined with a variation partitioning techniques to identify the relationships between environmental, spatial and temporal predictors structuring benthic metacommunities in estuarine systems. However, our prediction that benthic macroinfaunal metacommunities would follow a non-clumped associations characterized by a continual change in species composition along environmental gradients without the formation of discrete assemblages (i.e., Gleasonian distribution) was not supported. Instead, we found Q-nested and nested species composition with clumped species loss as the most frequent patterns. We also did not corroborate our prediction of the higher importance of pure environmental filters, but found a higher importance of the shared fraction between environment and space influencing benthic metacommunity. Therefore, in this system benthic communities along estuaries were generally subsets of a large pool of species (i.e. nested or Q-nested) and with space, salinity and sediment size were most strongly associated with this pattern.

Even though we observed six metacommunity types^7^, the overall metacommunity fitted Q-nested (Q-clumped species loss) or nested subsets (clumped species loss). Nestedness may arise when sites with lower species richness are subsets of richer sites as a result of environmental conditions of the habitats or species-specific characteristics, such as dispersal ability or tolerance of abiotic conditions^62^. Nested structures are not rare, and they have already been reported for aquatic metacommunities^10,15,20,41,63^. Most species found in estuarine systems have marine origin and diversification^23^, so the sites closer to the sea are richer while the sites closer to freshwater have poorer subsets, as fewer estuarine species can arrive and/or survive in such conditions (e.g., lower salinity and depth).

Some taxa, like polychaetes from the families Nereididae and Capitellidae, and also Tellinidae mollusks, showed a wide distribution along all estuarine systems (Fig. 4). However, Chironomidae (Insecta) and Oligochaeta, for example, are adapted to freshwater systems and occurred only at the sites farthest from the sea (Fig. 4). This partially explains nestedness being not so strong (Q-nested) and the distinct patterns found on different sampling occasions. Another important consideration is that, at timescales of months and years, the same taxa might migrate up or down the estuarine gradient in order to physiologically couple with environmental variability.

The emergence of a Q-Gleasonian gradient occurred only for one sampling period for the Subaé estuary. The upper zone (more freshwater) of this estuary is well known for its inorganic pollution in the sediments^64^.Given the high importance of the strong salinity gradient in estuarine systems, we expected the dominance of Gleasonian patterns at metacommunity level for all estuarine systems, as a result of species having differential responses to the environmental gradients. Nevertheless, it seems that a nestedness situation, where most of the taxa can live in more salty regions (richer sites) and some of them will tolerate different levels of freshwater, is predominant. The emergence of a Clementsian structure for one sampling period for the Paraguaçu estuary and a Q-Clementsian structure for two sampling periods for the Jaguaripe estuary was also unexpected. The Clementsian gradient may be related to historical biogeographic features such as the process of communities’ isolation and/or environmental variation^65^ showing that sets of species respond similarly to environmental variation, but the occurrence of environmental stochastic stress zones lead to clumped boundaries. Moreover, our study clearly shows that whenever metacommunity patterns are under investigation it is imperative to have replicates in time and space at landscape level (e.g., different estuaries, lakes, rivers etc sampled at different times) because such patterns are dynamic.

We observed a high amount of benthic metacommunity variation explained by the shared influence of environmental filters and spatial predictors for all estuaries. However, since the variation was not exclusively caused by spatial variables, we can still argue that environmental filters (i.e. salinity and sediment) are important in shaping benthic metacommunity structure^3^. Spatial predictors, measured as geographical distance, were considered to play a significant role in the similarity of species compositions between sites^1^. Accordingly, environmental variables in estuaries, especially salinity, were spatially structured, which may explain why the highest proportion of total variance was explained by the shared influence of environmental and spatial predictors (Fig. 5). Salinity decreases according to distance from sea to river (Fig. 3), and consequently may affect benthic metacommunity structure from high species and feeding-guild diversities to dominance by a single species or a feeding group^27^ and decrease in diversity at family level^21,22^.

Communities change dynamically in richness and composition over time, and consequently the underlying mechanisms are not static over time either^4,19,20,41^. Unlike the other systems, the Paraguaçu estuary had two sample campaigns in the same year representing two different seasons and it was the system in which variation partitioning showed temporal predictors as the most important component for explaining total variation. There is also a possibility that freshwater inflows into the Paraguaçu estuary may have greater variability than would naturally occur because of construction of the Pedra do Cavalo Dam during the 1980s and the implementation of the Pedra do Cavalo Hydroeletric Power Plant for energy generation in 2005^66^. Our results suggest that potential differences between those metacommunity structures (e.g. Paraguaçu Dec-2005 nested vs. Paraguaçu May-2005 Clementsian) and temporal components are worth additional research. We strongly suggest that future studies should include hypotheses explicitly related to temporal variation (i.e., seasons, drought/flood) with specific changes in metacommunity patterns.

For the same estuaries sampled in this study, Barros *et al.*^21^ found a decrease in diversity of benthic macroinfaunal assemblages, at family level, from marine to freshwater zones. Likewise, Barros *et al*.^22^ showed that α-diversity decreased along marine to freshwater conditions, while β-diversity was driven by replacement or nestedness depending on the level and distribution of disturbances in estuaries subjected to anthropogenic stressors. Both studies contradict the most popular estuarine model, the Remane model^28^, which suggests a diversity minimum zone called the *arteminimum*.

Contrastingly, environmental disturbances may result in environmental homogenization due to high dispersal, which can result in homogenization of metacommunities, increasing nestedness and decreasing species replacement^65,67^. The Subaé estuary is well-known to be impacted by human activity, with high levels of inorganic contaminants in the upper estuary^30^ and showed a nested pattern for four sampling periods out of five (Table 2). Jaguaripe and Paraguaçu estuaries also had a decrease in concentrations of contaminants seawards, but they are considered relatively well conserved. The less disturbed Jaguaripe estuary, displayed less nestedness (in two out of four sampling periods) than Paraguaçu (three out of four) (Table 2). Nestedness was more often found in the Subaé estuary, where the upper region (contaminated) is poorer in species richness compared to the lower region, likely accentuating the nestedness pattern from sea to freshwater.

There is some criticism about the EMS framework used to investigate idealized metacommunity patterns and especially whether the turnover test is adequate for detecting nested patterns, as turnover and nestedness are not necessarily mutually exclusive or opposite^38,45,46^. Nestedness can be measured using various metrics^26^, such as NODF, that may not be directly comparable to the one used in the context of the EMS framework. Consequently, results based on EMS to evaluate nestedness may be inconsistent if compared to these indices, which ordinate matrices based on richness of sites and species incidence. However, the reciprocal averaging method used in the EMS analysis for nested subsets discerns inter-site variation in response to a latent environmental gradient enhancing the association of mechanisms with nested structures and the form of species loss^7^. Also, if EMS are studied for a wide range of taxa and locations as in this study, general associations may emerge between particular idealized patterns of distribution and specific taxa^38^.

Since nestedness is among the non-random distribution patterns related to species-specific characteristics such as dispersal ability, habitat specialization and tolerance to abiotic conditions, future studies should integrate temporal dynamics, spatial predictors and environmental filters with dispersal traits and disturbances in the EMS approach. Dispersal mode is a regional process considered a strong driver for benthic metacommunity distribution pattern^4,11,12,19,67^ as more dispersive species are more controlled by the environment than less dispersive species^60^. Considering that many natural systems are subjected to human impacts, such as the upper region of the Subaé estuary, integrating the knowledge of how local (i.e., environmental filters, geographical distance) and regional (i.e., dispersal mode) processes structure natural systems may improve management.

Estuaries are strongly impacted by human activities, in addition to natural stressors, which can affect assemblage structure affecting the functioning of these important systems^26,68^. Knowing the type of structure and the drivers that shape metacommunities along different estuarine gradients and how that structure changes over time is important for future studies aiming at conservation to help to establish effective conservation policies^3,26^. Our study indicated that benthic metacommunities follow Q-nested and nested structures and are highly influenced by the shared influence of environmental and spatial predictors. We identified many common taxa occurring across all salinity gradients (e.g., Capitelllidae and Nereididae) as well as the most habitat-specialist taxa, with occurrence only in more freshwater sites (e.g., Chironomidae and Oligochaeta). More importantly, by studying a strong environmental gradient at different times we showed that metacommunity patterns will differ since environmental conditions will vary. We believe our study advances the knowledge on how estuarine benthic communities are structured. We showed that salinity and proximity (space) are major drivers and also highlighted the importance of spatial and temporal replication whenever investigating stronger ecological gradients. Future studies should incorporate functionality and explicit time-related hypotheses.

## Supplementary information

Electronic Supplementary Material.

Supplementary Dataset - Species Data.

Supplementary Dataset - Environmental Data.

Supplementary Dataset - Spatial Coordinates.
