# Supplementary material for: Drivers of benthic metacommunity structure along tropical estuaries
Source: Sci Rep. 2020 Feb 3;10:1739. doi: 10.1038/s41598-020-58631-1 (PMC6997391; doi:10.1038/s41598-020-58631-1)
Supplement: Supplementary file 1 — Electronic Supplementary Material. [file 41598_2020_58631_MOESM1_ESM.pdf]

Electronic Supplementary Material

Drivers of benthic metacommunity structure along tropical estuaries

Andreia Teixeira Alves[1*], Danielle Katharine Petsch[2], Francisco Barros[1]

*Corresponding author e-mail address: dea_alves106@yahoo.com.br ; Telephone: +55 71991542200

1. Laboratório de Ecologia Bentônica (LEB), Instituto de Biologia, Programa de Pós Graduação em Ecologia e Biomonitoramento, Universidade Federal da Bahia, Brazil

2. Núcleo de Pesquisas em Limnologia, Ictiologia e Aquicultura (Nupelia), Programa de Pós-Graduação em Ecologia de Ambientes Aquáticos Continentais (PEA), Universidade Estadual de Maringá, Brazil

Supplementary Figure 1 Sample-based rarefaction curves showing the relationship between sample effort (i.e., number of stations) and family richness for each estuarine system for the total sampling time

Supplementary Figure 2 Schematic representation used to examine how the EMS (i.e., coherence, turnover and boundary clumping) results in six main metacommunity structures (i.e., checkerboards, random, nested, evenly spaced, Gleasonian and Clementsian) and quasi-structures. Modified from Presley et al. (2010) and Brasil et al. (2017) for a) Subaé, b) Jaguaripe, and c) Paraguaçu; n= number of times the pattern was found

Supplementary Table 1 "Nestedness metric based on overlap and decreasing fill" (NODF) results and the significance values for each estuarine system

Supplementary Table 2 Variance inflation factor (VIF) values and the environmental predictors resulted from multicollinearity test for each system

Supplementary Table 3 Variation partitioning result for each estuarine system and the significance of environmental, spatial and temporal components individualistically

Supplementary Figure 1

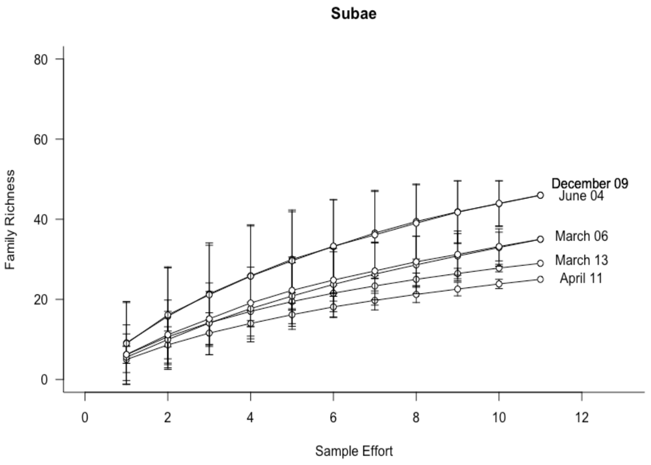

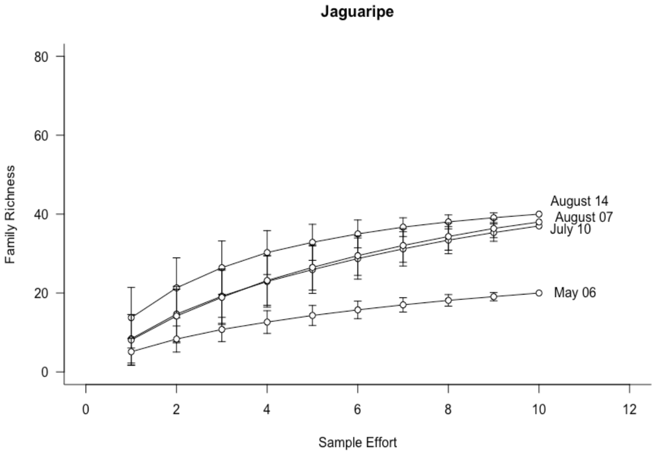

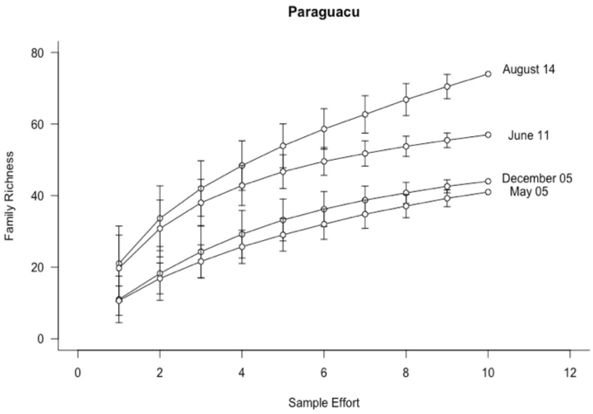

Supplementary Figure 2

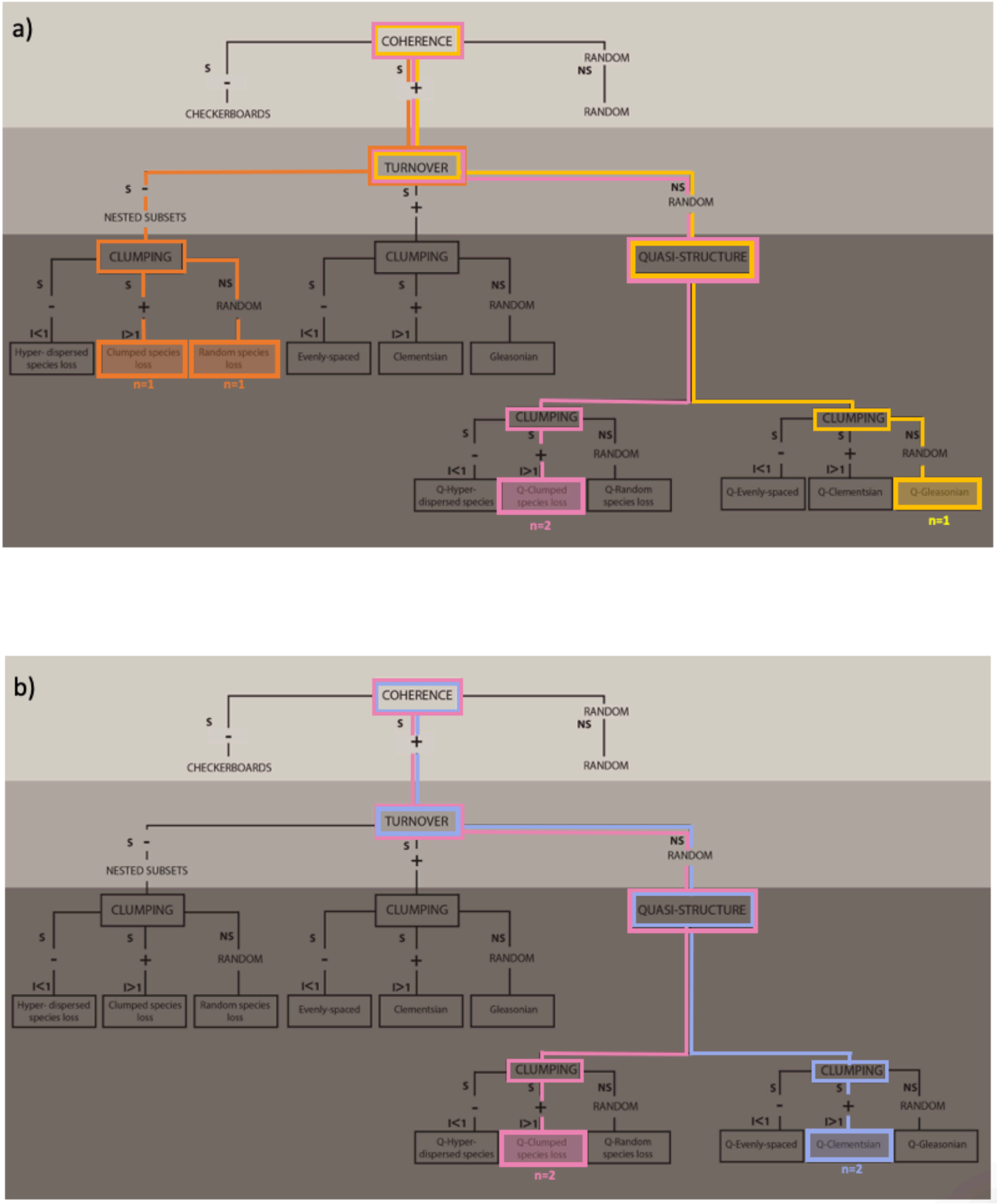

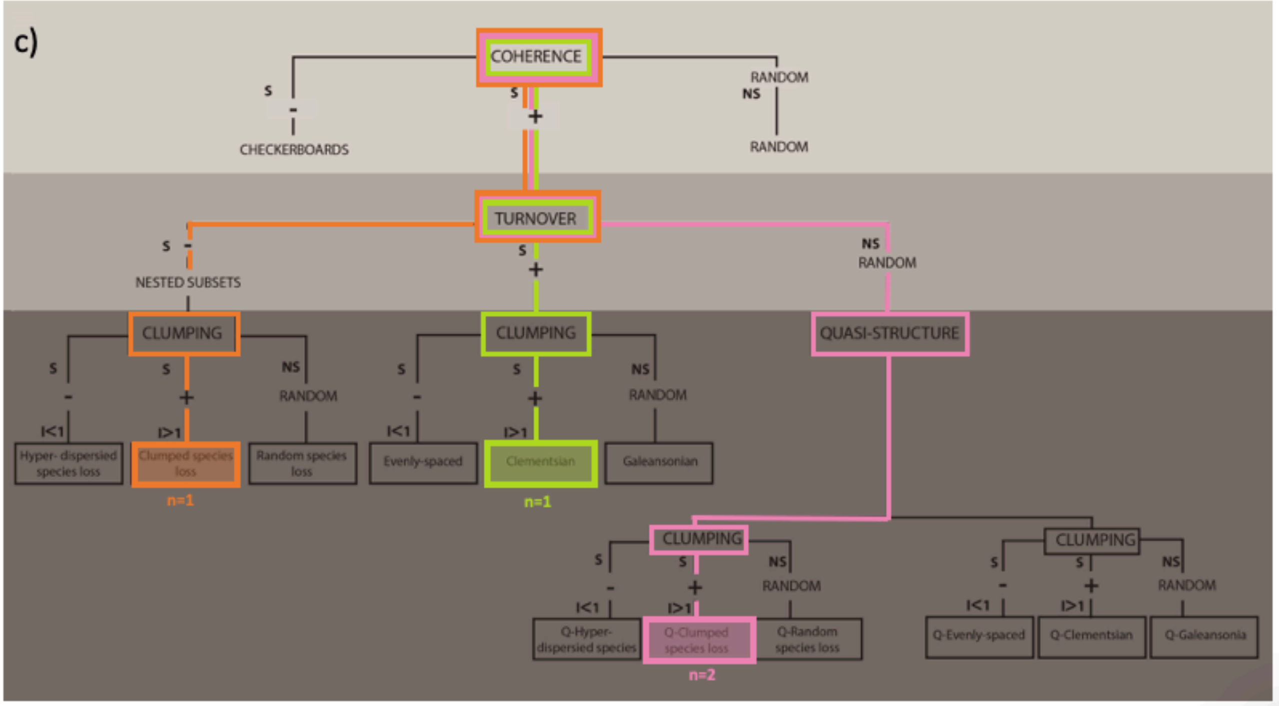
c)
COHERENCE
S
-
CHECKERBOARDS
S
+
RANDOM
NS
RANDOM
TURNOVER
S
-
NESTED SUBSETS
S
+
NS
RANDOM
CLUMPING
S
-
I<1
Hyper- dispersied species loss
S
+
I>1
Clumped species loss
n=1
NS
RANDOM
Random species loss
CLUMPING
S
-
I<1
Evenly-spaced
S
+
I>1
Clementsian
n=1
NS
RANDOM
Galeansonian
QUASI-STRUCTURE
CLUMPING
S
-
I<1
Q-Hyper-dispersied species
S
+
I>1
Q-Clumped species loss
n=2
NS
RANDOM
Q-Random species loss
CLUMPING
S
-
I<1
Q-Evenly-spaced
S
+
I>1
Q-Clementsian
NS
RANDOM
Q-Galeansonia

Supplementary Table 1 We performed "Nestedness metric based on overlap and decreasing fill" NODF. Prior to NODF, we generated a matrix of incidence (presence/absence of species for each sampling period and each estuary) by ordering sites into rows following salinity gradient (i.e., sites from sea to freshwater) and species occurrence into columns. We calculated NODF again as explained above, but pooling all samplings periods for each site in each estuary. (NODF) values among rows (Nrows), matrix fill and associated *P* values for each sampling period of Subaé, Jaguaripe and Paraguaçu estuaries. Significant *P* values are indicated by bold font

Estuary	Month	Year	Nrows	Matrix fill	*P*
Subaé	03	2013	20.82	0.050	**0.001**
Subaé	04	2011	44.83	0.041	**0.001**
Subaé	12	2009	36.39	0.069	**0.001**
Subaé	03	2006	28.10	0.045	**0.001**
Subaé	06	2004	55.44	0.049	**0.001**
Jaguaripe	08	2014	52.15	0.104	**0.001**
Jaguaripe	07	2010	44.92	0.066	**0.001**
Jaguaripe	08	2007	59.00	0.064	**0.001**
Jaguaripe	05	2006	63.48	0.041	**0.001**
Paraguaçu	08	2014	30.72	0.151	**0.001**
Paraguaçu	06	2011	37.76	0.146	**0.001**
Paraguaçu	12	2005	37.83	0.083	**0.001**
Paraguaçu	05	2005	41.89	0.081	**0.001**

(NODF) values among rows (Nrows), matrix fill and associated *P* values pooling all sampling periods for Subaé, Jaguaripe and Paraguaçu estuaries. Significant *P* values are indicated by bold font

Estuary	Nrows	Matrix fill	*P*
Subaé	48.22	0.140	**0.001**
Jaguaripe	40.25	0.276	**0.001**
Paraguaçu	60.94	0.147	**0.001**

Supplementary Table 2 Variance inflation factor (VIF) values and the remaining environmental predictors resulted from multicollinearity test after the removal of collinear explanatory variables characterized by with highest value (VIF >3) for each system

Estuarine system	Environmental predictors	Variance inflation factor
Subaé	Pebble	1.39
	Very coarse sand	1.67
	Medium sand	1.81
	Fine sand	1.29
	Very fine sand	1.61
	Salinity	1.72
Jaguaripe	Pebble	1.87
	Granule gravel	2.05
	Coarse sand	1.92
	Medium sand	1.50
	Very fine sand	1.47
	Salinity	1.70
Paraguaçu	Pebble	1.55
	Very coarse sand	1.59
	Medium sand	1.69
	Fine sand	1.78
	Very fine sand	1.69
	Salinity	2.21

Supplementary Table 3 Variation partitioning results showing the contribution of environmental variables [X1], spatial factors [X2] and temporal components [X3] to total variation of benthic assemblages for Subaé, Jaguaripe and Paraguaçu estuaries over time. The component [a] represents the purely environmental fraction, [b] purely spatial, [c] purely temporal, [d] shared environmental and spatial, [e] shared spatial and temporal, [f] shared environmental and temporal, [g] shared environmental, spatial and temporal components. Variance partitioning (%) and associated *P* values of the purely environmental, spatial and temporal components for each estuarine system

Subaé

**Component**	R^2	Adj.R^2
[a+d+f+g] = X1	0.20	0.13
[b+d+e+g] = X2	0.23	0.17
[c+e+f+g] = X3	0.10	0.03
[a+b+d+e+f+g] = X1+X2	0.32	0.20
[a+c+d+e+f+g] = X1+X3	0.29	0.17
[b+c+d+e+f+g] = X2+X3	0.33	0.21
[a+b+c+d+e+f+g] = All	0.40	0.23
**Individual fractions**		
[a] = X1 | X2+X3	0.02	
[b] = X2 | X1+X3	0.06	
[c] = X3 | X1+X2	0.03	
[d]	0.12	
[e]	0.10	
[f]	0.01	
[g]	-0.03	
[h] = Residuals	0.78	
**Controlling 1 table X**		
[a+d] = X1 | X3	0.15	
[a+f] = X1 | X2	0.03	
[b+d] = X2 | X3	0.19	
[b+e] = X2 | X1	0.07	
[c+e] = X3 | X1	0.04	
[c+f] = X3 | X2	0.04	

Purely environmental

Number of permutations:		999		
	Df	Variance	F	*P*
Model	4	0.07	1.57	0.003
Residual	45	0.50		

Purely spatial

Number of permutations:	999

	Df	Variance	F	*P*
Model	4	0.09	2.03	0.001
Residual	45	0.51		

Purely temporal

Number of permutations:	999			
	Df	Variance	F	*P*
Model	1	0.02	1.98	0.012
Residual	45	0.50		

Jaguaripe

**Component**	**R^2**	**Adj.R^2**
[a+d+f+g] = X1	0.33	0.25
[b+d+e+g] = X2	0.35	0.32
[c+e+f+g] = X3	0.14	0.07
[a+b+d+e+f+g] = X1+X2	0.48	0.38
[a+c+d+e+f+g] = X1+X3	0.47	0.35
[b+c+d+e+f+g] = X2+X3	0.49	0.41
[a+b+c+d+e+f+g] = All	0.58	0.45
**Individual fractions**		
[a] = X1 | X2+X3	0.04	
[b] = X2 | X1+X3	0.10	
[c] = X3 | X1+X2	0.08	
[d]	0.25	
[e]	0.03	
[f]	0.02	
[g]	-0.06	
[h] = Residuals	0.55	
**Controlling 1 table X**		
[a+d] = X1 | X3	0.29	
[a+f] = X1 | X2	0.06	
[b+d] = X2 | X3	0.35	
[b+e] = X2 | X1	0.13	
[c+e] = X3 | X1	0.10	
[c+f] = X3 | X2	0.10	

Purely environmental

Number of permutations:	999			
	Df	Variance	F	*P*
Model	4	0.07	1.57	0.017
Residual	32	0.33		

Purely spatial

Number of permutations:	999

	Df	Variance	F	*P*
Model	2	0.09	4.29	0.001
Residual	32	0.33		

Purely temporal

Number of permutations:		999		
	Df	Variance	F	*P*
Model	1	0.04	3.37	0.001
Residual	32	0.33		

Paraguaçu

**Component**	**R^2**	**Adj.R^2**
[a+d+f+g] = X1	0.14	0.10
[b+d+e+g] = X2	0.19	0.12
[c+e+f+g] = X3	0.19	0.12
[a+b+d+e+f+g] = X1+X2	0.28	0.17
[a+c+d+e+f+g] = X1+X3	0.35	0.25
[b+c+d+e+f+g] = X2+X3	0.38	0.27
[a+b+c+d+e+f+g] = All	0.44	0.30
**Individual fractions**		
[a] = X1 | X2+X3	0.03	
[b] = X2 | X1+X3	0.05	
[c] = X3 | X1+X2	0.13	
[d]	0.01	
[e]	0.03	
[f]	0.02	
[g]	-0.05	
[h] = Residuals	0.70	
**Controlling 1 table X**		
[a+d] = X1 | X3	0.13	
[a+f] = X1 | X2	0.05	
[b+d] = X2 | X3	0.14	
[b+e] = X2 | X1	0.08	
[c+e] = X3 | X1	0.16	
[c+f] = X3 | X2	0.15	

Purely environmental

Number of permutations:		999		
	Df	Variance	F	*P*
Model	3	0.05	1.75	0.001
Residual	33	0.43		

Purely spatial

Number of permutations:	999

	Df	Variance	F	*P*
Model	3	0.08	1.99	0.001
Residual	33	0.43		

Purely temporal

Number of permutations:		999		
	Df	Variance	F	*P*
Model	1	0.06	4.75	0.001
Residual	32	0.43		
