# Supplementary material for: Drivers of benthic metacommunity structure along tropical estuaries
Source: Sci Rep. 2020 Feb 3;10:1739. doi: 10.1038/s41598-020-58631-1 (PMC6997391; doi:10.1038/s41598-020-58631-1)
Supplement: Supplementary file 2 — Supplementary Dataset - Species Data. [file 41598_2020_58631_MOESM2_ESM.pdf]

Drivers of benthic metacommunity structure along tropical estuaries

Andreia Teixeira Alves[1*], Danielle Katharine Petsch[2], Francisco Barros[1]

*Corresponding author e-mail address: dea_alves106@yahoo.com.br ; Telephone: +55 71991542200 1.

1. Laboratório de Ecologia Bentônica (LEB), Instituto de Biologia, Programa de Pós Graduação em Ecologia e Biomonitoramento, Universidade Federal da Bahia, Brazil

2. Núcleo de Pesquisas em Limnologia, Ictiologia e Aquicultura (Nupelia), Programa de Pós- Graduação em Ecologia de Ambientes Aquáticos Continentais (PEA), Universidade Estadual de Maringá, Brazil

Supplementary Dataset Species Data

Estuary	Month	Year	Sites	Arabellidae
Subaé	March	2013	#1	0
Subaé	March	2013	#2	0
Subaé	March	2013	#3	0
Subaé	March	2013	#4	0
Subaé	March	2013	#5	0
Subaé	March	2013	#6	0
Subaé	March	2013	#7	0
Subaé	March	2013	#8	0
Subaé	March	2013	#9	0
Subaé	March	2013	#10	0
Subaé	March	2013	#11	0
Subaé	April	2011	#1	0
Subaé	April	2011	#2	0
Subaé	April	2011	#3	0
Subaé	April	2011	#4	0
Subaé	April	2011	#5	0
Subaé	April	2011	#6	0
Subaé	April	2011	#7	0
Subaé	April	2011	#8	0
Subaé	April	2011	#9	0
Subaé	April	2011	#10	0
Subaé	April	2011	#11	0
Subaé	December	2009	#1	0
Subaé	December	2009	#2	0
Subaé	December	2009	#3	0
Subaé	December	2009	#4	0
Subaé	December	2009	#5	0
Subaé	December	2009	#6	0
Subaé	December	2009	#7	0

Subaé	December	2009	#8	0
Subaé	December	2009	#9	0
Subaé	December	2009	#10	0
Subaé	December	2009	#11	0
Subaé	March	2006	#1	0
Subaé	March	2006	#2	1
Subaé	March	2006	#3	0
Subaé	March	2006	#4	0
Subaé	March	2006	#5	0
Subaé	March	2006	#6	0
Subaé	March	2006	#7	0
Subaé	March	2006	#8	0
Subaé	March	2006	#9	0
Subaé	March	2006	#10	0
Subaé	March	2006	#11	0
Subaé	June	2004	#1	0
Subaé	June	2004	#2	0
Subaé	June	2004	#3	0
Subaé	June	2004	#4	0
Subaé	June	2004	#5	0
Subaé	June	2004	#6	0
Subaé	June	2004	#7	0
Subaé	June	2004	#8	0
Subaé	June	2004	#9	0
Subaé	June	2004	#10	0
Subaé	June	2004	#11	0
Jaguaripe	August	2014	#1	0
Jaguaripe	August	2014	#2	0
Jaguaripe	August	2014	#3	0
Jaguaripe	August	2014	#4	0
Jaguaripe	August	2014	#5	0
Jaguaripe	August	2014	#6	0
Jaguaripe	August	2014	#7	0
Jaguaripe	August	2014	#8	0
Jaguaripe	August	2014	#9	0
Jaguaripe	August	2014	#10	0
Jaguaripe	August	2010	#1	0
Jaguaripe	August	2010	#2	0
Jaguaripe	August	2010	#3	0
Jaguaripe	August	2010	#4	0
Jaguaripe	August	2010	#5	0
Jaguaripe	August	2010	#6	0
Jaguaripe	August	2010	#7	0

Jaguaripe	August	2010	#8	0
Jaguaripe	August	2010	#9	0
Jaguaripe	August	2010	#10	0
Jaguaripe	August	2007	#1	0
Jaguaripe	August	2007	#2	0
Jaguaripe	August	2007	#3	0
Jaguaripe	August	2007	#4	0
Jaguaripe	August	2007	#5	0
Jaguaripe	August	2007	#6	0
Jaguaripe	August	2007	#7	0
Jaguaripe	August	2007	#8	0
Jaguaripe	August	2007	#9	0
Jaguaripe	August	2007	#10	0
Jaguaripe	May	2006	#1	0
Jaguaripe	May	2006	#2	0
Jaguaripe	May	2006	#3	0
Jaguaripe	May	2006	#4	0
Jaguaripe	May	2006	#5	0
Jaguaripe	May	2006	#6	0
Jaguaripe	May	2006	#7	0
Jaguaripe	May	2006	#8	0
Jaguaripe	May	2006	#9	0
Jaguaripe	May	2006	#10	0
Paraguaçu	August	2014	#1	0
Paraguaçu	August	2014	#2	0
Paraguaçu	August	2014	#3	0
Paraguaçu	August	2014	#4	0
Paraguaçu	August	2014	#5	0
Paraguaçu	August	2014	#6	0
Paraguaçu	August	2014	#7	0
Paraguaçu	August	2014	#8	0
Paraguaçu	August	2014	#9	0
Paraguaçu	August	2014	#10	0
Paraguaçu	June	2011	#1	0
Paraguaçu	June	2011	#2	0
Paraguaçu	June	2011	#3	0
Paraguaçu	June	2011	#4	0
Paraguaçu	June	2011	#5	0
Paraguaçu	June	2011	#6	0
Paraguaçu	June	2011	#7	0
Paraguaçu	June	2011	#8	0
Paraguaçu	June	2011	#9	0
Paraguaçu	June	2011	#10	0

Paraguaçu	May	2005	#1	0
Paraguaçu	May	2005	#2	0
Paraguaçu	May	2005	#3	0
Paraguaçu	May	2005	#4	0
Paraguaçu	May	2005	#5	0
Paraguaçu	May	2005	#6	0
Paraguaçu	May	2005	#7	0
Paraguaçu	May	2005	#8	0
Paraguaçu	May	2005	#9	0
Paraguaçu	May	2005	#10	0
Paraguaçu	December	2005	#1	0
Paraguaçu	December	2005	#2	0
Paraguaçu	December	2005	#3	0
Paraguaçu	December	2005	#4	0
Paraguaçu	December	2005	#5	0
Paraguaçu	December	2005	#6	0
Paraguaçu	December	2005	#7	0
Paraguaçu	December	2005	#8	0
Paraguaçu	December	2005	#9	0
Paraguaçu	December	2005	#10	0

**Cirratulidae**	**Glyceridae**	**Goniadidae**	**Lacydoniidae**	**Magelonidae**
1	0	0	0	0
0	0	0	0	0
0	0	1	0	0
1	0	0	0	0
0	1	1	0	0
0	0	1	0	1
0	1	1	0	0
0	0	0	0	0
0	0	0	0	0
0	0	1	0	0
0	0	0	0	0
0	1	0	0	0
0	1	1	0	0
1	0	1	0	0
0	1	1	0	1
0	1	1	0	0
0	1	1	0	0
0	0	0	0	0
0	0	0	0	0
0	0	0	0	0
0	0	0	0	0
0	0	0	0	0

0	1	0	0	0
1	0	1	0	1
0	0	0	0	0
1	0	0	0	0
0	0	1	0	1
0	1	0	0	1
1	0	1	0	1
0	0	0	0	0
0	0	0	0	0
0	0	0	0	0
0	0	0	0	0
1	0	0	1	0
1	0	0	0	1
0	1	0	0	0
1	0	1	0	0
0	0	0	0	0
0	0	1	0	1
0	1	0	0	1
0	0	0	0	0
0	0	0	0	0
0	0	0	0	0
0	0	0	0	0
0	0	1	0	0
1	0	0	0	0
0	0	0	0	0
0	0	1	0	0
0	0	0	0	0
0	0	1	0	0
0	0	1	0	0
0	0	0	0	0
0	0	0	0	0
0	0	0	0	0
0	0	0	0	0
1	0	1	0	0
1	1	1	0	0
1	1	1	0	1
0	0	1	0	1
0	1	1	0	1
0	0	1	0	0
0	0	0	0	0
0	0	0	0	0
0	0	0	0	0
0	0	0	0	0

1	1	0	0	0
1	0	1	0	1
0	0	1	0	1
0	0	0	0	1
0	0	1	0	1
0	0	0	0	0
0	0	0	0	0
0	0	0	0	0
0	0	0	0	0
0	0	0	0	0
1	1	0	0	1
1	1	1	0	1
1	0	1	0	1
1	1	0	0	1
0	1	0	0	1
0	1	0	0	0
0	1	0	0	0
0	0	0	0	0
0	0	0	0	0
0	0	0	0	0
1	0	0	0	1
1	1	0	0	1
1	1	0	0	1
0	0	1	0	1
0	0	0	0	1
0	1	0	0	1
0	0	1	0	0
0	0	0	0	0
0	0	0	0	0
0	0	0	0	0
0	0	0	0	0
1	1	0	0	1
1	0	1	0	1
1	1	0	0	0
1	1	1	0	1
1	1	1	0	1
0	1	1	0	1
0	1	1	0	1
0	1	0	0	0
0	1	1	0	0
1	0	1	0	0
1	1	1	0	1
1	1	0	0	0

1	1	0	0	0
1	1	0	0	1
1	1	0	0	1
1	1	1	0	1
0	1	0	0	0
0	1	0	0	0
0	0	0	0	0
1	0	0	0	0
1	1	0	0	0
1	0	0	0	0
0	0	0	0	0
1	0	0	0	0
1	0	0	0	0
0	1	0	0	0
0	0	0	0	0
0	0	0	0	0
0	0	0	0	0
1	0	0	0	0
1	1	0	0	0
1	1	0	0	0
1	0	0	0	0
0	1	0	0	0
1	1	0	0	0
0	1	0	0	0
0	1	0	0	0
0	1	0	0	0
0	1	0	0	0

**Nereididae**	**Orbiniidae**	**Pilargidae**	**Poecilochaetidae**	**Spionidae**
0	0	0	0	0
0	0	0	0	0
1	0	0	0	0
1	0	0	0	0
1	0	0	0	0
1	0	0	0	0
0	1	1	0	0
0	1	1	0	1
1	0	1	0	0
1	0	1	0	1
1	0	0	0	0
0	1	0	0	0
0	1	0	0	0
1	1	1	0	0
1	1	1	0	0

0	1	0	0	1
0	1	1	0	0
0	1	1	0	0
0	0	0	0	0
0	0	0	0	0
1	0	0	0	0
0	0	0	0	0
0	1	0	0	0
1	1	0	0	1
0	0	0	0	0
0	0	1	0	1
0	1	0	0	0
1	1	0	0	0
1	1	1	0	1
0	0	1	0	1
0	0	0	0	0
1	0	0	0	1
1	0	0	0	1
0	1	0	0	1
0	1	0	0	1
0	0	0	0	0
0	1	0	0	0
0	0	0	0	1
0	1	0	0	1
0	1	0	1	0
0	1	0	0	0
0	0	0	0	0
1	0	1	0	1
1	0	1	0	0
0	0	0	0	1
1	1	0	0	0
0	0	0	0	0
0	0	0	1	0
0	0	0	0	0
0	1	0	0	0
0	0	0	0	0
0	0	0	0	0
1	0	0	0	0
1	0	0	0	0
0	0	0	0	0
0	1	1	0	1
1	1	1	1	1
1	1	1	1	1

0	0	1	0	1
0	0	1	0	0
1	0	1	1	1
1	0	1	0	1
1	0	1	0	1
1	0	0	0	1
1	0	0	0	0
0	1	0	1	1
1	1	1	1	1
1	0	0	0	0
0	0	0	0	0
0	0	1	0	0
0	1	0	0	0
0	0	0	0	1
1	0	1	0	0
1	0	0	1	1
1	0	0	0	0
1	1	0	1	0
0	1	0	0	1
1	1	0	0	0
1	1	0	1	0
0	1	0	0	0
0	0	1	0	0
1	0	1	0	1
1	0	0	0	0
1	0	0	0	0
1	0	0	0	0
0	1	0	0	0
1	1	0	0	0
1	1	0	1	0
0	1	1	1	0
0	1	0	0	0
0	1	0	0	0
0	1	0	0	0
1	0	1	0	0
1	0	0	0	0
0	0	0	0	0
0	0	0	0	0
1	1	1	1	1
1	1	1	0	1
1	1	0	1	1
1	1	0	1	1
1	0	1	1	1

1	1	1	0	1
1	0	1	0	1
0	1	0	0	0
0	0	1	0	1
1	1	0	0	1
1	1	1	1	1
1	0	1	1	1
1	1	0	0	1
1	1	0	1	1
1	1	1	1	1
1	1	1	0	1
0	0	0	0	1
1	0	0	0	1
0	0	0	0	1
1	0	1	0	0
1	0	0	0	0
1	0	0	0	0
1	1	0	0	1
1	0	0	1	0
1	0	0	1	0
1	0	0	0	0
1	0	0	0	0
1	0	0	0	0
1	0	0	0	0
1	0	0	0	0
1	1	0	0	0
0	0	0	1	0
0	0	0	0	0
0	1	0	0	0
1	0	0	1	1
0	0	0	0	0
0	0	0	0	0
0	0	0	0	0
0	0	0	0	0
**Syllidae**	**Veneridae**	**Lucinidae**	**Corbulidae**	**Tellinidae**
0	0	1	0	1
0	0	0	0	0
0	0	0	0	0
0	0	0	0	1
0	0	0	0	0
0	0	0	0	0
0	0	0	0	1
0	0	0	0	1

0	0	0	0	1
0	0	0	0	0
0	0	0	0	0
0	0	0	0	1
0	0	0	0	0
0	0	0	1	0
0	0	0	1	1
0	0	0	0	0
0	0	0	0	0
0	0	0	0	1
0	0	0	0	1
0	0	0	0	0
0	0	0	0	1
0	0	0	0	0
0	0	0	1	1
1	1	0	1	1
0	0	0	0	0
1	0	0	1	0
0	0	0	0	0
0	0	0	0	0
0	1	0	0	0
1	0	0	0	1
0	0	0	0	1
1	0	0	0	1
0	0	0	0	1
0	0	0	0	0
1	1	1	1	0
0	0	0	0	0
0	0	0	0	0
0	0	0	0	0
0	0	0	0	0
0	0	0	0	1
0	0	0	0	1
0	0	0	0	1
0	0	0	0	0
0	0	0	0	0
0	0	0	1	1
1	1	0	1	0
1	0	0	0	0
1	0	0	1	0
0	0	0	0	0
1	0	0	0	1
0	0	0	0	1

0	0	0	0	1
0	0	0	0	0
0	0	0	0	0
0	0	0	0	0
0	1	0	0	1
1	0	0	0	1
1	0	0	0	1
0	0	0	0	0
0	0	0	0	1
0	0	0	0	1
0	0	0	0	1
0	0	0	0	1
0	0	0	0	0
0	0	0	0	0
0	1	0	0	1
0	0	0	1	1
0	0	0	0	0
0	0	0	0	0
0	0	0	0	1
0	0	0	0	1
0	0	0	0	1
0	0	0	0	1
0	0	1	0	0
0	0	0	0	0
0	1	0	0	1
0	0	0	0	0
0	1	0	0	1
0	1	0	0	0
0	0	0	0	1
0	0	0	0	1
0	0	0	0	1
0	0	0	0	0
0	0	0	0	0
0	0	0	0	0
0	0	0	0	0
0	0	0	0	1
1	0	0	0	1
0	0	0	0	1
0	0	0	0	1
0	0	0	0	1
0	0	0	0	1
0	1	0	0	1
0	0	0	0	0

0	0	0	0	0
0	0	0	0	0
0	1	0	1	1
0	1	0	1	0
1	0	0	1	0
0	1	0	1	1
0	1	1	1	1
0	1	0	0	1
0	0	0	1	1
0	0	0	0	1
0	0	0	0	1
0	1	0	0	1
1	1	0	0	1
0	0	0	0	1
1	0	0	0	1
1	1	1	0	1
1	1	0	0	1
0	1	0	0	1
0	0	0	0	1
0	1	0	0	1
0	0	0	0	1
0	0	0	0	1
0	0	1	0	1
0	0	0	0	1
1	0	0	0	1
0	0	1	0	1
0	1	1	1	1
0	0	1	0	1
0	0	0	0	1
0	0	0	0	1
0	0	0	0	1
0	0	0	1	1
0	0	0	1	1
0	0	0	0	1
0	1	0	1	1
0	0	1	0	1
1	0	1	0	1
0	1	0	0	1
0	1	0	0	1
0	1	0	0	1
0	1	0	0	1
**Mactridae**	**Mytillidae**	**Psammobiidae**	**Sipuncula**	**Columbellidae**
0	0	0	0	0

0	0	1	0	0
0	0	0	0	0
0	0	0	1	0
0	0	0	0	0
0	0	0	1	0
0	1	0	1	0
0	1	0	1	0
0	0	0	0	0
0	0	0	0	0
0	0	0	0	0
0	0	0	0	0
0	0	0	0	0
0	0	0	0	0
0	0	1	0	0
0	0	0	0	0
0	0	0	0	0
0	0	1	0	0
0	1	0	0	0
0	0	0	0	0
0	0	0	0	0
0	0	0	0	0
0	0	0	0	0
0	0	0	1	1
0	0	0	0	0
0	0	0	0	0
0	0	0	0	0
0	0	0	0	0
0	0	0	0	0
0	0	0	0	0
0	0	0	0	0
0	0	0	0	0
0	0	0	0	0
0	0	0	0	0
1	0	0	1	1
0	0	0	0	0
0	0	0	0	0
0	0	0	0	0
0	0	0	0	0
0	0	1	0	0
0	0	0	0	0
0	1	0	0	0
0	0	0	0	0
0	0	1	0	0

0	0	0	0	0
0	0	0	0	0
0	0	0	0	0
0	0	0	0	0
0	0	0	0	0
0	0	0	0	0
0	0	0	0	0
0	0	0	0	0
0	0	0	0	0
0	0	0	0	0
0	0	0	0	0
0	0	0	1	0
0	0	0	1	0
0	0	0	1	0
0	0	0	1	0
0	0	0	1	0
0	0	0	1	0
0	0	0	0	0
0	0	0	0	0
0	0	0	0	0
0	0	0	0	0
0	0	0	0	0
0	0	0	0	0
0	0	0	1	0
0	0	0	1	0
0	0	0	0	0
0	0	0	1	0
0	0	0	0	0
0	0	0	0	0
0	0	0	1	0
0	0	0	0	0
0	0	0	0	0
0	0	1	0	0
0	0	0	0	0
0	0	0	0	0
0	0	0	0	0
0	0	0	0	0
0	0	0	0	0
0	0	0	0	0
0	0	0	0	0
0	0	0	0	0
0	0	0	1	0
0	0	0	0	0

0	0	0	0	0
0	0	0	0	0
0	0	0	0	0
0	0	0	0	0
0	0	0	0	0
0	0	0	0	0
0	0	0	0	0
0	0	0	0	0
0	0	0	0	0
1	0	0	1	0
0	0	1	1	0
0	0	0	1	0
0	0	1	0	0
0	0	0	1	0
1	0	0	1	0
0	0	0	0	1
0	0	0	1	0
0	0	0	1	0
1	0	0	0	0
1	0	0	1	0
1	0	0	1	0
1	0	0	0	0
1	0	0	1	0
1	0	0	1	0
0	0	0	1	0
0	0	0	0	0
0	0	0	1	0
0	0	0	0	0
0	0	0	1	1
0	0	0	1	0
0	0	0	0	0
0	0	0	0	0
0	0	0	0	0
0	0	1	0	0
0	0	0	0	0
0	0	0	0	0
0	0	0	0	0
0	0	0	0	0
0	0	0	0	0
0	0	0	0	0
0	0	0	0	0
0	0	0	0	0
0	0	0	0	0

0	0	1	0	0
0	0	0	0	0
0	0	0	0	0
0	0	0	0	0
0	0	1	0	0
**Vitrinellidae**	**Ischnochitonidae**	**Xanthidae**	**Paguridae**	**Branchiostomidae**
0	0	0	0	0
0	0	0	0	0
0	0	0	0	0
0	0	0	0	0
0	0	0	0	0
0	0	0	0	0
0	0	0	0	1
0	0	0	0	0
0	0	0	0	0
0	0	0	0	0
0	0	0	0	0
0	0	0	0	0
0	0	0	0	0
0	0	0	0	0
0	0	0	0	0
0	0	0	0	0
0	0	0	0	0
0	0	0	0	0
0	0	0	0	0
0	0	0	0	0
0	0	0	0	0
0	0	0	0	0
0	0	0	0	0
0	0	0	0	0
0	1	0	0	0
0	0	0	0	0
0	0	0	0	0
0	0	0	0	0
0	0	0	0	0
0	0	0	0	0
0	0	0	0	0
0	0	0	0	0
0	0	0	0	0
0	0	0	0	0
0	0	0	0	0
0	1	1	1	0
1	0	0	0	0
0	0	0	0	0

0	0	0	0	0
0	0	0	0	0
0	0	0	0	1
0	0	0	0	0
0	0	0	0	0
0	0	0	0	0
0	0	0	0	0
0	0	0	0	0
0	0	0	0	0
0	0	0	0	0
0	0	0	0	0
0	0	0	0	0
0	0	0	1	0
0	0	0	0	0
0	0	0	0	0
0	0	0	0	0
0	0	0	0	0
0	0	0	0	0
0	0	0	0	1
0	0	0	0	1
0	0	0	0	1
0	0	0	0	0
0	0	0	0	0
0	0	0	0	0
0	0	0	0	0
0	0	0	0	0
0	0	0	0	0
0	0	0	0	0
0	0	0	0	1
0	0	1	0	1
0	0	0	0	0
0	0	0	0	0
0	0	0	0	0
0	0	0	0	0
0	0	0	0	0
0	0	0	0	0
0	0	0	0	0
0	0	0	0	0
0	0	0	0	1
0	0	0	1	1
0	0	0	0	0
0	0	0	0	0
0	0	0	1	0

0	0	0	0	0
0	0	0	0	0
0	0	0	0	0
0	0	0	0	0
0	0	0	0	0
0	0	0	0	1
0	0	0	0	1
0	0	0	0	1
0	0	0	0	0
0	0	0	0	0
0	0	0	0	0
0	0	0	0	0
0	0	0	0	0
0	0	0	0	0
0	0	0	0	0
0	0	0	0	0
0	0	0	0	0
0	0	0	0	0
0	0	0	1	0
0	0	0	0	1
0	0	0	0	0
0	0	0	0	0
0	0	0	0	0
0	0	0	0	0
0	0	0	0	0
0	0	0	0	0
0	0	0	0	0
0	0	0	0	0
0	0	1	0	0
0	0	1	0	0
0	0	1	0	0
0	0	0	0	0
0	0	1	0	0
0	0	0	0	0
0	0	0	0	0
0	0	0	0	0
0	0	0	0	0
0	0	0	1	0
0	0	0	1	0
0	0	0	1	0
0	0	0	1	0
0	0	0	0	0
0	0	0	0	0

0	0	0	0	0
0	0	0	0	0
0	0	0	0	0
0	0	0	0	0
0	0	0	1	0
0	0	0	1	0
0	0	0	0	0
0	0	0	1	1
0	0	0	0	0
0	0	0	0	0
0	0	0	0	0
0	0	0	0	0
**Eunicidae**	**Myidae**	**Noetiidae**	**Nuculidae**	**Onuphidae**
0	0	0	0	0
0	0	0	0	0
0	0	0	0	0
0	0	0	0	0
0	0	0	0	1
0	0	0	0	0
0	0	0	0	1
0	0	0	0	0
0	0	0	0	0
0	0	0	0	0
0	0	0	0	0
0	0	0	0	0
0	0	0	0	0
0	0	0	0	0
0	0	0	0	0
0	0	0	0	1
0	0	0	0	0
0	0	0	0	0
0	0	0	0	0
0	0	0	0	0
0	0	0	0	0
0	0	0	0	0
0	0	0	0	0
0	0	0	0	1
0	0	0	0	0
0	0	0	0	0
0	0	0	0	0
0	0	0	0	0
0	0	0	0	0
0	0	0	0	0

0	0	0	0	0
0	0	0	0	0
0	0	0	0	0
0	0	0	0	0
0	0	0	0	0
0	0	0	0	0
0	0	0	0	0
0	0	0	0	0
0	0	0	0	0
0	0	0	0	0
0	0	0	0	0
0	0	0	0	0
0	0	0	0	0
0	0	0	0	0
0	0	0	0	0
1	0	0	0	1
0	0	0	0	0
0	0	0	0	0
0	0	0	0	0
1	0	0	0	1
0	0	0	0	0
0	0	0	0	0
0	0	0	0	0
0	0	0	0	0
0	0	0	0	0
1	0	0	0	1
1	0	0	0	1
1	0	0	0	1
0	0	0	0	0
0	0	0	0	0
0	0	0	0	0
0	0	0	0	0
0	0	0	0	0
0	0	0	0	0
0	0	0	0	0
0	0	0	0	0
1	0	0	0	1
0	0	0	0	1
0	0	0	0	0
0	0	0	0	0
0	0	0	0	0
0	0	0	0	0
0	0	0	0	0

0	0	0	0	0
0	0	0	0	0
0	0	0	1	1
0	0	0	0	1
0	0	0	0	0
0	0	0	0	0
0	0	0	0	0
0	0	0	0	0
0	0	0	0	0
0	0	0	0	0
0	0	0	0	0
0	0	0	0	0
0	0	0	0	0
1	0	0	0	0
0	0	0	0	1
0	0	0	0	0
0	0	0	0	0
0	0	0	0	0
0	0	0	0	0
0	0	0	0	0
0	0	0	0	0
0	0	0	0	0
0	0	0	0	0
0	0	0	0	1
0	0	0	0	1
0	0	0	1	1
0	0	0	0	1
1	0	0	0	1
0	0	0	1	0
0	0	0	0	0
0	0	0	0	0
0	0	0	0	0
0	0	0	1	0
0	0	0	1	1
0	0	0	0	1
0	0	0	1	0
1	0	0	1	1
0	0	0	0	1
0	0	0	0	0
0	0	0	0	0
0	0	0	0	0
0	0	0	0	0
0	0	1	1	0

0	0	0	1	1
0	0	0	1	0
0	0	0	1	0
0	0	0	1	1
1	1	0	1	1
0	0	0	1	0
0	0	0	0	0
0	0	0	0	0
0	0	0	0	0
0	0	0	1	0
0	0	0	1	0
0	0	0	1	0
0	0	0	0	0
0	1	1	0	0
0	1	0	0	0
0	0	0	0	0
0	0	0	0	0
0	0	0	0	0
0	0	0	0	0
**Stenopodidea**	**Paraonidae**	**Phyllodocidae**	**Sabellidae**	**Terebellidae**
0	1	0	0	0
0	0	0	0	0
0	0	0	0	0
0	1	0	0	0
0	0	0	0	0
0	1	0	0	0
0	0	0	0	0
0	0	0	0	0
0	0	0	0	0
0	0	0	0	0
0	1	0	0	0
0	0	0	0	0
0	0	0	0	0
0	0	0	0	0
0	0	0	0	0
0	0	0	0	0
0	0	0	0	0
0	0	0	0	0
0	0	0	0	0
0	0	0	0	0
0	0	0	0	0
0	0	0	0	0
0	0	0	0	0

0	1	0	1	1
0	0	0	0	1
0	0	0	0	0
0	0	0	0	1
0	0	0	0	0
0	0	0	0	0
0	0	0	0	0
0	0	0	0	0
0	0	0	0	0
0	0	0	0	0
0	0	0	0	0
1	0	0	0	0
1	0	0	0	0
0	0	0	0	0
0	0	0	0	0
0	0	0	0	0
0	0	0	0	0
0	0	0	0	0
0	0	0	0	0
0	0	0	0	0
0	0	0	0	0
0	1	0	0	0
0	0	1	1	1
0	0	0	0	0
0	0	0	0	0
0	0	0	0	0
0	0	0	0	0
0	0	0	0	0
0	0	0	0	0
0	0	0	0	0
0	0	0	0	0
0	0	0	0	0
0	1	0	0	0
0	1	0	0	0
0	1	1	0	0
0	1	0	0	0
0	1	0	0	0
0	0	1	0	0
0	0	0	0	0
0	0	0	0	0
0	0	0	0	0
0	0	0	0	0
0	1	0	0	0

0	1	0	1	0
0	1	0	0	0
0	1	0	0	0
0	1	0	0	0
0	1	1	0	0
0	0	0	0	0
0	0	0	0	0
0	0	0	0	0
0	0	0	0	0
0	0	1	0	0
0	0	0	0	0
0	0	0	0	0
0	0	0	0	1
0	0	1	0	0
0	0	0	0	0
0	0	0	0	0
0	0	0	0	0
0	0	0	0	0
0	0	0	0	0
0	0	0	0	0
0	0	0	0	0
0	0	0	0	0
0	0	0	0	0
0	0	0	0	0
0	0	0	0	0
0	0	0	0	0
0	0	0	0	0
0	0	0	0	0
0	0	0	0	0
0	0	0	0	0
0	1	0	0	0
0	1	0	0	0
0	0	0	0	0
0	1	1	0	0
0	1	0	0	1
0	1	0	0	0
0	0	0	0	0
0	1	0	0	0
0	0	0	0	0
0	0	0	0	0
0	1	0	0	0
0	1	1	0	0
0	0	0	0	0

0	1	1	1	0
0	1	1	0	0
0	1	1	0	0
0	0	0	0	0
0	0	0	0	0
0	0	0	0	0
0	0	0	1	0
0	0	0	0	0
0	0	0	0	0
0	0	0	0	0
0	0	0	0	0
0	0	1	0	0
0	0	0	0	0
0	0	0	0	0
0	0	0	0	0
0	0	0	0	0
0	0	0	0	0
0	0	0	1	0
0	0	0	0	0
0	0	0	0	0
0	0	0	1	1
0	0	0	0	1
0	0	0	0	0
0	0	1	0	0
0	0	0	0	0
0	0	0	0	0
**Cnidaria**	**Porifera**	**Lumbrineridae**	**Solecurtidae**	**Ostreidea**
0	0	0	0	0
0	0	0	0	0
0	0	0	0	0
0	0	0	0	0
0	0	0	0	0
0	0	0	0	0
0	0	0	0	0
0	0	0	0	0
0	0	0	0	0
0	0	0	1	0
0	0	0	0	0
0	0	0	0	0
0	0	0	0	0
0	0	0	0	0
0	0	0	0	0
0	0	0	0	0

0	0	0	0	0
0	0	0	0	0
0	0	0	0	0
0	0	0	0	0
0	0	0	0	0
0	0	0	0	0
0	0	0	0	0
0	0	1	0	1
0	0	0	0	0
0	0	0	0	0
0	0	0	0	0
0	0	0	1	0
0	0	0	1	0
0	0	0	0	0
0	0	0	0	0
0	0	0	0	0
0	0	0	0	0
0	0	0	0	0
1	1	0	0	0
0	0	0	0	0
0	0	0	0	0
0	1	0	0	0
0	0	0	0	0
0	0	0	0	0
0	0	0	0	0
0	0	0	0	0
0	0	0	0	0
0	0	0	0	0
0	0	1	1	0
1	0	1	0	1
0	0	0	1	0
0	0	0	1	0
0	0	0	0	0
1	0	0	1	0
0	0	0	0	0
0	0	0	0	0
0	0	0	0	0
0	0	0	0	0
0	0	0	0	0
0	0	1	0	0
0	0	1	0	0
0	0	1	0	0
0	0	0	0	0

0	0	0	0	0
0	0	0	0	0
0	0	0	0	0
0	0	0	0	0
0	0	0	0	0
0	0	0	0	0
0	0	0	0	0
0	0	1	0	0
0	0	0	0	0
0	0	0	0	0
0	0	0	0	0
0	0	0	0	0
0	0	0	0	0
0	0	0	1	0
0	0	0	0	0
0	0	0	0	0
1	0	0	0	0
1	1	0	0	1
0	0	0	0	0
0	0	0	0	0
0	0	0	0	0
0	0	0	0	0
0	0	0	0	1
0	0	0	0	0
0	0	0	0	0
0	0	0	0	0
0	0	0	0	0
0	0	0	0	0
0	0	0	0	0
0	0	0	0	0
0	0	0	0	0
0	0	0	0	0
0	0	0	0	0
0	0	0	0	0
0	0	0	0	0
0	0	0	0	0
0	0	0	0	0
0	0	1	0	0
0	0	1	0	0
0	0	0	0	0
0	0	0	0	0
0	0	0	1	0
0	0	0	1	0

0	0	0	0	0
0	0	0	0	0
0	0	0	0	0
0	0	0	0	0
0	0	1	0	0
0	0	1	0	0
0	0	1	0	0
0	0	1	0	0
0	0	0	0	0
0	0	0	0	0
0	0	0	1	0
0	0	0	0	0
0	0	0	1	0
0	0	0	0	0
1	0	0	0	0
0	0	0	0	0
1	0	0	0	0
1	0	0	0	0
1	0	0	0	0
0	0	0	0	0
0	0	0	0	1
0	0	0	0	1
0	0	0	0	0
1	1	0	0	0
1	0	0	0	0
0	0	0	0	0
0	0	0	0	0
0	1	0	0	1
1	1	1	0	0
0	1	0	0	0
0	0	0	0	1
0	0	0	0	0
0	0	0	0	0

**Ostracoda**	**Hesionidae**	**Nephtyidae**	**Bryozoa**	**Ampharetidae**
0	0	0	0	0
0	0	0	0	0
0	0	0	0	0
0	0	0	0	0
0	0	0	0	0
0	0	0	0	0
0	0	0	0	0
0	0	0	0	0
0	0	0	0	0

0	0	0	0	1
0	0	0	0	0
0	0	0	0	0
0	0	0	0	0
0	0	0	0	0
0	0	0	0	0
0	0	0	0	0
0	0	0	0	0
0	0	0	0	0
0	0	0	0	0
0	0	0	0	0
0	0	0	0	0
0	0	0	0	0
0	0	0	0	0
0	0	0	0	0
1	0	0	0	0
0	0	0	0	0
0	0	0	0	0
0	0	0	0	0
0	0	0	0	0
0	0	0	0	0
0	0	0	0	0
0	0	0	0	0
0	0	0	0	1
0	0	0	0	0
0	0	0	0	0
0	0	0	0	0
0	0	0	0	0
0	0	0	0	0
0	0	0	0	0
0	0	0	0	0
0	0	0	0	0
0	0	0	0	0
0	0	0	0	0
0	0	0	0	0
1	0	0	0	0
0	0	0	1	0
0	0	0	0	0
0	0	0	0	0
0	0	0	0	0
0	0	0	1	0
0	0	0	0	0
0	0	0	0	0

0	0	0	0	0
0	0	0	0	0
0	0	0	0	0
1	0	0	0	0
0	0	0	0	0
0	0	0	0	1
0	0	0	0	0
0	0	0	0	0
0	0	0	0	0
0	0	0	0	0
0	0	0	0	0
1	0	0	0	0
1	0	0	0	0
0	0	0	0	0
0	0	0	0	1
0	0	0	0	0
0	0	0	0	0
0	0	0	0	0
0	0	0	0	0
0	0	0	0	0
0	0	0	0	0
0	0	0	0	0
0	0	0	0	0
0	0	1	0	1
0	0	0	0	0
0	0	0	0	0
0	0	0	0	0
0	0	0	0	0
0	0	0	0	0
0	0	0	0	0
0	0	0	0	0
0	0	0	0	0
0	0	0	0	0
0	0	0	0	0
0	0	0	0	0
0	0	0	0	0
0	0	0	0	0
0	0	0	0	0
0	0	0	0	0
0	0	0	0	0
0	0	0	0	0
0	0	0	0	0
0	0	0	0	0

0	0	0	0	0
1	0	0	0	0
1	0	0	0	0
0	0	0	0	1
0	0	0	0	0
0	0	0	0	0
1	0	0	0	0
0	0	0	0	0
0	0	0	0	0
0	0	0	0	0
0	0	0	0	0
0	0	0	0	0
0	0	0	0	0
0	0	0	0	0
1	0	0	0	0
0	0	0	0	0
0	0	0	0	0
0	0	0	0	0
0	0	0	0	0
1	0	0	0	0
0	0	0	1	0
0	0	0	1	0
1	0	0	1	0
0	0	0	1	1
0	0	0	1	1
0	1	0	1	0
0	1	0	0	0
0	0	0	1	1
0	0	0	1	0
0	0	0	0	0
0	0	0	1	0
0	0	0	1	0
0	0	0	1	0
0	0	0	0	0
0	0	0	1	0
0	0	0	0	0
0	1	0	0	0
0	0	0	0	0
0	0	0	0	0
0	0	0	0	0
**Paralacydoniidae**	**Turbonillidae**	**Tanaidaceo**	**Gammaridea**	**Callianassidae**
0	0	0	0	0
0	0	0	0	0

0	0	0	0	0
0	0	0	0	0
0	0	0	0	0
0	0	0	0	0
0	0	0	0	0
0	0	0	0	0
0	0	0	0	0
0	0	0	0	0
0	0	0	0	0
0	0	0	0	0
0	0	0	0	0
0	0	0	0	0
0	0	0	0	0
0	0	0	0	0
0	0	0	0	0
0	0	0	0	0
0	0	0	0	0
0	0	0	0	0
0	0	0	0	0
0	0	0	0	0
0	0	0	0	0
0	0	0	1	0
0	0	0	0	0
0	0	0	0	0
0	0	0	0	0
0	0	0	0	0
0	0	0	0	0
0	0	0	0	0
0	0	0	0	0
0	0	0	0	0
0	0	0	0	0
0	0	0	0	0
0	0	0	0	0
0	0	0	0	0
0	0	0	0	0
0	0	0	0	0
0	0	0	0	0
0	0	0	0	0
0	0	0	0	0
0	0	0	0	0
0	0	0	0	0
0	0	0	0	0
0	0	1	1	0

0	1	0	0	1
0	0	0	0	0
0	0	0	0	0
0	0	0	0	0
0	0	0	0	0
0	0	0	0	0
0	0	0	0	0
0	0	0	0	0
0	0	0	0	0
0	0	0	0	0
0	0	1	0	0
0	0	1	0	0
0	0	1	0	0
0	0	0	0	0
0	0	0	0	0
0	0	0	0	0
0	0	0	0	0
0	0	0	0	0
0	0	0	0	0
0	0	0	0	0
0	0	1	0	0
0	0	0	0	0
0	0	0	0	0
0	0	0	0	0
0	0	0	0	0
0	0	0	0	0
0	0	0	0	0
0	0	0	0	0
0	0	0	0	0
0	0	0	0	0
0	0	0	0	0
0	0	0	0	0
1	0	0	0	0
1	0	0	0	0
1	0	0	0	0
0	0	0	0	0
0	0	0	0	0
0	0	0	0	0
0	0	0	1	0
0	0	0	0	0
0	0	0	0	0
0	0	0	0	0
0	0	0	0	0

0	0	0	0	0
0	0	0	0	0
0	0	0	0	0
0	0	0	0	0
0	0	0	0	0
0	0	0	0	0
0	0	0	0	0
0	0	0	0	0
0	0	1	1	0
0	0	0	0	0
0	0	0	1	0
0	0	1	0	0
0	0	1	0	0
0	0	0	0	0
0	0	0	0	0
0	0	0	0	0
0	0	1	0	0
0	0	0	0	0
0	0	1	0	0
0	0	0	0	0
0	0	0	0	0
0	0	0	1	1
1	0	0	0	0
0	0	0	0	0
0	0	0	0	0
0	0	0	1	0
0	0	0	0	0
0	0	0	0	0
0	0	0	0	0
0	0	0	0	0
0	0	0	0	0
0	0	0	0	0
0	0	0	0	0
0	0	0	0	0
0	0	0	0	0
0	0	0	0	0
0	0	0	0	0
0	0	0	0	0
0	0	0	0	0
0	0	0	0	0
0	0	0	0	0
0	0	0	0	0
0	0	0	1	0

0	0	0	0	0
0	0	0	0	0
0	0	0	0	0
0	0	0	0	0
**Arcidae**	**Olividae**	**Corophiidae**	**Nemertea**	**Anthuridae**
0	0	0	0	0
0	0	0	0	0
0	0	0	0	0
0	0	0	0	0
0	0	0	0	0
0	0	0	0	0
0	0	0	0	0
0	0	0	0	0
0	0	0	0	0
0	0	0	0	0
0	0	0	0	0
0	0	0	0	0
0	0	0	0	0
1	0	0	0	0
0	0	0	1	0
0	0	0	0	0
0	0	0	0	0
0	0	0	1	0
0	0	0	0	0
0	0	0	0	0
0	0	0	0	0
0	0	0	0	0
1	0	0	1	0
1	0	0	1	1
0	0	0	0	0
1	0	0	0	0
1	0	0	0	0
0	0	0	0	0
0	0	0	0	0
0	0	0	0	0
0	0	0	0	0
0	0	0	0	0
0	0	0	0	0
0	0	0	0	0
0	0	0	0	0
0	0	0	0	0
0	0	0	0	0
0	0	0	0	0

0	0	0	0	0
0	0	0	0	0
0	0	0	0	0
0	0	0	0	0
0	0	0	0	0
0	0	0	0	0
0	0	0	0	0
0	0	1	1	0
0	0	0	0	0
0	0	0	1	0
0	0	0	0	0
0	0	0	0	0
0	0	0	1	0
0	0	0	0	0
0	0	0	0	0
0	0	0	0	0
0	0	0	0	0
0	0	0	1	0
0	0	0	1	0
0	0	0	1	0
0	0	0	1	0
0	0	0	0	0
0	0	0	1	0
0	0	0	1	0
0	0	0	1	0
0	0	0	1	0
0	0	0	1	0
0	1	0	0	1
0	0	0	0	0
0	0	0	0	0
0	0	0	0	0
0	0	0	0	0
0	0	0	0	0
0	0	0	0	0
0	0	0	0	0
0	0	0	0	0
0	0	0	0	0
1	0	0	0	1
0	0	0	0	1
0	0	1	0	1
0	0	0	0	0
0	0	0	1	1
0	0	0	1	0

0	0	0	0	0
0	0	0	0	0
0	0	0	0	0
0	0	0	0	0
0	1	0	0	1
0	0	0	0	0
0	0	0	0	0
0	0	0	0	0
0	0	0	0	0
0	0	0	0	0
0	0	0	0	0
0	0	0	0	0
0	0	0	0	0
0	0	0	0	0
0	0	0	0	0
0	0	0	0	0
0	0	0	1	0
0	0	0	0	0
0	0	0	1	0
0	0	0	1	0
0	0	0	1	0
0	0	0	0	0
0	0	0	0	0
0	0	0	1	0
0	0	0	1	0
1	0	0	1	0
0	0	0	1	0
0	0	0	0	0
1	0	0	1	0
0	1	0	1	0
0	0	0	1	0
0	0	0	1	0
0	0	0	1	0
0	0	0	0	0
0	0	0	0	0
0	0	0	0	0
0	0	0	0	0
0	0	0	0	0
0	0	0	0	0
0	0	0	0	0
0	0	0	0	0
0	0	0	0	0
0	0	0	0	0

0	0	0	0	0
0	0	0	0	0
0	0	0	0	0
0	0	0	0	0
0	0	0	0	0
0	0	0	0	0
0	0	0	0	0
0	0	0	0	0
0	0	0	0	0
0	0	0	0	0
0	0	0	0	0

Caecidae	Nuculanidae	Eulimidae	Ophiactidae	Ophiothrichidae
0	1	0	0	0
0	0	0	0	0
0	0	0	0	0
0	1	0	0	0
0	0	0	0	0
0	0	0	0	0
0	0	0	0	0
0	0	0	0	0
0	0	0	0	0
0	0	0	0	0
0	0	0	0	0
0	0	0	0	0
0	0	0	0	0
0	0	0	0	0
0	0	0	0	0
0	0	0	0	0
0	0	0	0	0
0	0	0	0	0
0	0	0	0	0
0	0	0	0	0
0	0	0	0	0
0	0	0	0	0
0	0	0	0	0
0	0	0	0	0
0	0	0	0	0
0	0	0	0	0
0	0	0	0	0
0	0	0	0	0
0	0	0	0	0
0	0	0	0	0
0	0	0	0	0

0	0	0	0	0
0	0	0	0	0
0	0	0	0	0
0	0	0	0	0
0	0	0	0	0
0	0	0	0	0
0	0	0	0	0
0	0	0	0	0
0	0	0	0	0
0	0	0	0	0
0	0	0	0	0
0	0	0	0	0
0	0	0	0	0
0	0	0	0	0
0	0	0	0	0
0	0	0	0	0
0	0	0	0	0
0	0	0	0	0
0	0	0	0	0
0	0	0	0	0
0	0	0	0	0
0	0	0	0	0
0	0	0	0	0
0	0	0	0	0
1	0	0	0	0
0	0	0	0	0
0	0	0	0	0
0	0	0	0	0
0	0	0	0	0
0	0	0	0	0
0	0	0	0	0
0	0	0	0	0
0	0	0	0	0
0	0	0	0	0
0	0	0	0	0
0	0	0	0	0
0	0	0	0	0
0	0	0	0	0
0	0	0	0	0
0	0	0	0	0
0	0	0	0	0
0	0	0	0	0
0	0	0	0	0

0	0	0	0	0
0	0	0	0	0
1	0	0	0	0
0	0	0	0	0
0	0	1	0	0
0	0	0	0	0
0	0	0	0	0
0	0	0	0	0
0	0	0	0	0
0	0	0	0	0
0	0	0	0	0
0	0	0	0	0
0	0	0	0	0
0	0	0	0	0
0	0	0	0	0
0	0	0	0	0
0	0	0	0	0
0	0	0	0	0
0	0	0	0	0
0	0	0	0	0
0	0	0	0	0
0	0	0	0	0
0	0	0	0	0
0	0	0	0	0
0	0	0	0	0
0	0	0	0	0
0	0	0	0	0
0	0	0	0	0
0	0	0	0	0
0	0	0	0	0
0	0	0	0	0
0	0	0	0	0
0	0	1	0	0
0	0	0	0	0
0	0	0	0	0
0	0	0	0	0
0	0	0	0	0
0	0	0	0	0
0	0	0	0	0
0	0	0	0	0
0	0	0	0	0
0	0	0	1	0
0	0	0	0	0

0	0	0	0	0
0	0	0	0	0
0	0	0	0	0
0	0	0	0	0
0	0	0	0	0
0	0	0	0	0
0	0	0	0	0
0	0	0	1	0
0	0	0	0	0
0	1	0	0	0
0	0	0	0	0
0	0	0	0	0
0	0	0	1	0
0	0	0	0	1
0	0	0	0	0
0	0	0	0	0
0	0	0	0	0
0	0	0	0	0

**Amphinomidae**	**Pectinariidae**	**Polynoidae**	**Cirolanidae**	**Penaeoidea**
0	0	0	0	0
0	0	0	0	0
0	0	0	0	0
0	0	0	0	0
0	0	0	0	0
0	0	0	0	0
0	0	0	0	0
0	0	0	0	0
0	0	0	0	0
0	0	0	0	0
0	0	0	0	0
0	0	0	0	0
0	0	1	0	0
0	0	1	0	0
0	0	0	0	0
0	0	0	0	0
0	0	0	0	0
0	0	0	0	0
0	0	0	0	0
0	0	0	0	0
0	0	0	0	0
0	0	0	0	0
0	0	1	0	1
0	1	1	0	1

0	0	0	0	1
0	0	0	0	1
0	0	0	0	0
0	0	0	0	0
0	0	0	0	0
0	0	0	0	0
0	0	0	0	0
0	0	0	0	0
0	0	0	0	0
0	0	0	0	0
0	0	0	0	0
0	0	0	0	0
0	0	0	0	0
0	0	0	0	0
0	0	0	0	0
0	0	0	0	0
0	0	0	0	0
0	0	0	0	0
0	0	0	0	0
0	0	0	0	0
0	0	0	0	0
0	0	0	0	1
0	0	0	0	0
0	0	0	0	1
0	0	0	0	0
0	0	0	0	1
0	0	0	0	0
0	0	0	0	0
0	0	0	0	0
0	0	0	0	0
0	0	0	0	0
0	0	0	0	0
0	0	0	0	0
0	0	0	0	0
0	0	0	0	0
0	0	0	0	0
0	0	0	0	0
0	0	0	0	0
0	0	0	0	0
0	0	0	0	0
0	0	0	0	0
0	0	0	0	0
0	0	0	0	0

0	0	0	0	0
0	0	0	0	0
0	0	0	0	0
0	0	0	0	0
0	0	0	0	0
0	0	0	0	0
0	0	0	0	0
0	0	0	0	0
0	0	0	0	0
0	0	0	0	0
0	0	0	0	0
0	0	0	0	0
0	0	0	0	0
0	0	0	0	0
0	0	0	0	0
0	0	0	0	0
0	0	0	0	0
0	0	0	0	0
0	0	0	0	0
0	0	0	0	0
0	0	0	0	0
0	0	0	0	0
0	0	0	0	0
0	0	0	0	0
0	0	0	0	0
0	0	0	0	0
0	0	0	0	0
0	0	0	0	0
0	0	0	0	0
0	0	1	0	0
0	0	0	0	0
0	0	0	0	0
0	0	1	0	0
0	0	1	0	0
0	0	0	0	0
0	0	0	1	0
0	0	0	1	0
0	0	0	1	0
3	0	1	0	0
0	3	1	0	0
0	1	1	0	0
0	2	1	0	0
0	1	1	0	0

1	0	1	0	0
0	0	0	0	0
0	0	0	1	0
0	0	0	1	0
0	0	0	1	0
0	0	1	0	0
0	0	0	0	1
0	1	0	0	0
0	0	0	0	1
0	0	0	0	0
0	0	0	0	1
0	0	0	0	1
0	0	1	0	0
0	0	0	1	1
0	0	0	1	0
0	0	0	0	1
1	1	0	0	1
0	0	1	0	1
0	0	0	0	0
0	0	1	0	0
1	1	0	0	1
0	0	0	0	0
0	0	0	1	0
0	0	0	0	0
0	0	0	1	0
**Grapsidae**	**Cyclopoida**	**Calappidae**	**Nannasticidae**	**Decapoda**
0	0	0	0	0
0	0	0	0	0
0	0	0	0	0
0	0	0	0	0
0	0	0	0	0
0	0	0	0	0
0	0	0	0	0
0	0	0	0	0
0	0	0	0	0
0	0	0	0	0
0	0	0	0	0
0	0	0	0	0
0	0	0	0	0
0	0	0	0	0
0	0	0	0	0
0	0	0	0	0
0	0	0	0	0

0	0	0	0	0
0	0	0	0	0
0	0	0	0	0
0	0	0	0	0
0	0	0	0	0
0	0	0	0	0
0	0	0	0	1
0	0	0	0	0
0	0	0	0	0
0	0	0	0	1
0	0	0	0	0
0	0	0	0	0
0	0	0	0	0
0	0	0	0	0
0	0	0	0	0
0	0	0	0	0
0	0	0	0	0
0	0	0	0	0
0	0	0	0	0
0	0	0	0	0
0	0	0	0	0
0	0	0	0	0
0	0	0	0	0
0	0	0	0	0
0	0	0	0	0
0	0	0	0	0
0	0	0	0	0
0	0	0	0	0
0	0	0	0	1
0	0	0	0	0
0	0	0	0	0
0	0	0	0	0
0	0	0	0	0
0	0	0	0	0
0	0	0	0	0
0	0	0	0	0
0	0	0	0	0
0	0	0	0	0
0	0	0	0	0
0	0	0	0	0
0	0	0	0	0
0	0	0	0	0
0	0	0	0	0

0	0	0	0	0
0	0	0	0	0
0	0	0	0	0
0	0	0	0	0
0	0	0	0	0
0	0	0	0	0
0	0	0	0	0
0	0	0	0	0
0	0	0	0	0
0	0	0	0	0
0	0	0	0	0
0	0	0	0	0
0	0	0	0	0
0	0	0	0	0
0	0	0	0	0
0	0	0	0	0
0	0	0	0	0
0	0	0	0	0
0	0	0	0	0
0	0	0	0	0
0	0	0	0	0
0	0	0	0	0
0	0	0	0	0
0	0	0	0	0
0	0	0	0	0
0	0	0	0	0
0	0	0	0	0
0	0	0	0	0
0	0	0	0	0
0	0	0	0	0
0	0	0	0	0
0	0	0	0	0
0	0	0	0	0
0	0	0	0	0
0	0	0	0	0
0	0	0	0	0
0	0	0	0	0
0	0	0	0	0
0	0	0	0	0
0	0	0	0	0
0	0	0	0	0
0	0	0	0	0
0	0	0	0	0

0	0	0	0	0
0	0	0	0	1
0	0	0	0	0
0	0	0	0	0
0	0	0	0	0
0	0	0	0	0
0	0	0	0	0
0	0	0	0	0
0	1	0	0	0
0	1	0	0	0
0	1	0	0	0
0	0	0	0	0
1	0	1	0	0
0	0	0	0	0
0	0	0	0	0
0	0	0	0	0
0	0	0	0	0
1	0	0	0	0
0	0	0	0	0
0	1	0	0	0
0	0	0	0	0
0	1	0	0	0
1	0	0	0	0
1	0	0	0	0
0	0	0	0	0
0	0	0	0	0
0	0	0	0	0
1	0	0	0	0
0	0	0	0	0
0	0	0	0	0
0	0	0	1	0
0	0	0	0	0

**Ophiuroidea**	**Alpheidae**	**Caprellidae**	**Cardiidae**	**Dorvilleidae**
0	0	0	0	0
0	0	0	0	0
0	0	0	0	0
0	0	0	0	0
0	0	0	0	0
0	0	0	0	0
0	0	0	0	0
0	0	0	0	0
0	0	0	0	0

0	0	0	0	0
0	0	0	0	0
0	0	0	0	0
0	0	0	0	1
0	0	0	0	0
0	1	0	0	0
0	0	0	1	0
0	0	0	0	0
0	0	0	0	0
0	0	0	0	0
0	0	0	0	0
0	0	0	0	0
0	0	0	0	0
0	0	0	0	0
1	0	1	5	1
0	0	0	0	0
0	1	0	0	0
0	0	0	0	0
0	0	0	0	0
0	0	0	0	0
0	0	0	0	0
0	0	0	0	0
0	0	0	0	0
0	0	0	0	0
0	0	0	0	0
0	0	0	0	0
0	0	0	0	0
0	0	0	0	0
0	0	0	0	0
0	0	0	0	0
0	0	0	0	0
0	0	0	0	0
0	0	0	0	0
0	0	0	0	0
0	0	0	0	0
0	0	0	0	0
0	0	0	0	0
0	0	0	0	0
0	0	0	0	0
0	0	0	0	0
0	0	0	0	0
0	0	0	0	0
0	0	0	0	0

0	0	0	0	0
0	0	0	0	0
0	0	0	0	0
0	0	0	0	1
1	0	0	0	0
0	0	0	0	0
0	0	0	0	0
0	0	0	0	0
0	0	0	0	0
0	0	0	0	0
0	0	0	0	0
0	0	0	0	0
0	0	0	0	0
1	0	0	1	0
1	0	0	1	0
0	0	0	0	0
0	0	0	0	0
0	0	0	0	0
0	0	0	0	0
0	0	0	0	0
0	0	0	0	0
0	0	0	0	0
0	0	0	0	0
1	0	0	0	0
0	0	0	0	0
0	0	0	0	0
0	0	0	0	0
0	0	0	0	0
0	0	0	0	0
0	0	0	0	0
0	0	0	0	0
0	0	0	0	0
0	0	0	0	0
0	0	0	0	0
0	0	0	0	0
0	0	0	0	0
0	0	0	0	0
0	0	0	0	0
0	0	0	0	0
0	0	0	0	0
0	0	0	0	0
0	0	0	0	0
0	0	0	0	0

0	0	0	0	0
1	0	0	0	0
0	0	0	0	0
1	0	0	0	1
1	0	0	0	0
1	1	0	0	1
0	0	0	0	1
0	0	0	0	1
0	0	0	0	0
0	0	0	0	0
1	0	0	0	0
1	0	0	0	0
1	0	0	0	1
1	0	0	0	1
1	0	3	0	1
0	0	0	0	0
0	0	0	0	1
0	0	0	0	0
0	0	0	0	0
0	0	0	0	0
0	0	0	0	0
0	0	0	0	0
0	0	0	0	0
0	0	0	0	0
0	0	0	0	0
0	0	0	0	0
0	0	0	0	0
0	0	0	0	0
0	0	0	0	0
0	0	0	0	0
0	0	0	0	0
0	0	0	0	0
0	0	0	0	0
0	0	0	0	0
0	0	0	0	0
0	0	0	0	0
0	0	0	0	0
0	0	0	0	0
0	0	0	0	0
0	0	0	0	0
0	0	0	0	0
**Pycnogonida**	**Pyramidellidae**	**Semelidae**	**Trichobranchidae**	**Aoridae**
0	0	0	0	0
0	0	0	0	0

0	0	0	0	0
0	0	0	0	0
0	0	0	0	0
0	0	0	0	0
0	0	0	0	0
0	0	0	0	0
0	0	0	0	0
0	0	0	0	0
0	0	0	0	0
0	0	0	1	0
0	0	0	0	0
0	0	0	0	0
0	0	0	0	0
0	0	0	0	0
0	0	0	0	0
0	0	0	0	0
0	0	0	0	0
0	0	0	0	0
0	0	0	0	0
0	0	0	0	0
0	0	0	0	0
1	1	1	1	0
0	0	0	0	0
0	0	0	0	0
0	0	0	0	0
0	0	0	0	0
0	0	1	0	0
0	0	0	0	0
0	0	0	0	0
0	0	0	0	0
0	0	0	0	0
0	0	0	0	0
0	0	0	0	0
0	0	0	0	0
0	0	0	0	0
0	0	0	0	0
0	0	0	0	0
0	0	0	0	0
0	0	0	0	0
0	0	0	0	0
0	0	0	0	0
0	0	0	0	0
0	0	0	0	0

0	0	0	0	0
0	0	0	0	0
0	0	0	0	0
0	0	0	0	0
0	0	0	0	0
0	0	0	0	0
0	0	0	0	0
0	0	0	0	0
0	0	0	0	0
0	0	0	0	0
0	0	0	0	0
0	0	1	1	0
0	0	1	1	0
0	0	1	0	0
0	0	0	0	0
0	0	0	0	0
0	0	0	0	0
0	0	0	0	0
0	0	0	0	0
0	0	1	0	0
0	0	0	0	0
0	0	0	0	0
0	0	0	0	0
0	0	0	0	0
0	0	0	0	0
0	0	0	0	0
0	0	0	0	0
0	0	0	0	0
0	0	0	0	0
0	0	0	0	0
0	0	0	0	0
0	0	0	0	0
0	0	0	0	0
0	0	0	0	0
0	0	0	0	0
0	0	0	0	0
0	0	0	0	0
0	0	0	0	0
0	0	0	0	0
0	0	0	0	0
0	0	0	0	0
0	0	0	0	0
0	0	0	0	0

0	0	0	0	0
0	0	0	0	0
0	0	0	0	0
0	0	0	0	0
0	0	0	0	0
0	0	0	0	0
0	0	0	0	0
0	0	0	0	0
0	1	0	0	0
0	0	1	0	0
0	0	0	0	0
0	0	0	1	1
0	0	0	1	0
0	0	1	0	1
0	0	0	0	0
0	0	0	0	1
0	0	0	0	0
0	0	1	0	0
0	0	1	1	0
0	0	0	0	0
0	0	1	0	0
0	0	1	1	0
0	0	1	1	0
0	0	0	1	0
0	0	0	0	0
0	0	0	0	0
0	0	0	0	0
0	0	0	0	0
0	0	0	0	0
0	0	0	0	0
0	0	0	0	0
0	0	0	0	0
0	0	0	0	0
0	0	0	0	0
0	0	0	0	0
0	0	0	0	0
0	0	0	0	0
0	0	0	0	0
0	0	0	0	0
0	0	0	0	0
0	0	0	0	0
0	0	0	0	0
0	0	0	0	0

0	0	0	0	0
0	0	0	0	0
0	0	0	0	0
0	0	0	0	0
**Glycymerididae**	**Ocypodidae**	**Amphilochidae**	**ascidia**	**Oligochaeta**
0	0	0	0	0
0	0	0	0	0
0	0	0	0	0
0	0	0	0	0
0	0	0	0	0
0	0	0	0	0
0	0	0	0	0
0	0	0	0	0
0	0	0	0	0
0	0	0	0	0
0	0	0	0	1
0	0	0	0	0
0	0	0	0	0
0	0	0	0	0
0	0	0	0	0
0	0	0	0	0
0	0	0	0	0
0	0	0	0	0
0	0	0	0	0
0	0	0	0	0
0	0	0	0	0
0	0	0	0	0
0	0	0	0	0
0	0	0	0	0
0	0	0	0	0
0	0	0	0	0
0	0	0	0	0
0	0	0	0	0
0	0	0	0	0
0	0	0	0	0
0	0	0	0	0
0	0	0	0	0
0	0	0	0	0
0	0	0	0	0
0	0	0	0	0
0	0	0	0	0
0	0	0	0	0
0	0	0	0	0

0	0	0	0	0
0	0	0	0	0
0	0	0	0	0
0	0	0	0	0
0	0	0	0	0
0	0	0	0	0
0	0	0	0	0
0	0	0	0	0
0	0	0	0	0
0	0	0	0	0
0	0	0	0	0
0	0	0	0	0
0	0	0	0	0
0	0	0	0	0
0	0	0	0	0
0	0	0	0	0
0	0	0	0	0
0	0	0	0	0
0	0	0	0	0
1	0	0	0	0
0	0	0	0	0
0	0	0	0	0
0	0	0	0	1
0	0	0	0	0
0	0	0	0	0
0	0	0	0	1
0	0	0	0	0
0	0	0	0	0
0	0	0	0	0
0	0	0	0	0
0	0	0	0	0
0	0	0	0	0
0	0	0	0	0
0	0	0	0	0
0	0	0	0	0
0	0	0	0	0
0	0	0	0	0
0	0	0	0	0
0	0	0	0	0
0	0	0	0	0
0	0	0	0	0
0	0	0	0	0
0	0	0	0	0

0	0	0	0	0
0	0	0	0	0
0	0	0	0	0
0	0	0	0	0
0	0	0	0	0
0	0	0	0	0
0	0	0	0	0
0	0	0	0	0
0	0	0	0	0
0	0	0	0	0
0	0	0	0	0
0	0	0	0	0
0	0	0	0	0
0	0	0	0	0
0	0	0	0	0
0	0	0	0	0
0	0	0	0	0
0	0	0	0	0
0	0	0	0	0
0	0	0	0	1
0	0	0	0	0
0	0	0	0	1
0	0	0	0	1
0	0	0	0	1
0	0	0	0	0
0	0	0	0	0
0	0	0	0	0
0	0	0	0	0
0	0	0	0	0
0	0	0	0	0
0	0	0	0	0
0	0	0	0	0
0	0	0	0	0
0	0	0	0	1
0	0	0	0	0
0	0	0	0	0
0	0	0	0	0
0	0	0	0	0
0	0	0	0	0
0	0	0	0	0
0	0	0	0	0
0	0	0	0	0
0	0	0	0	0

0	0	0	0	0
0	0	0	0	0
0	0	0	0	0
0	0	0	0	0
0	0	0	0	0
0	0	0	0	0
0	0	0	0	0
0	0	0	0	0
0	0	0	0	0
0	0	0	0	0
0	0	0	0	0
**Porcelanidae**	**AMPELISCIDAE**	**GONEPLACIDAE**	**ANOMURA**	**Peneidae**
0	0	0	0	0
0	0	0	0	0
0	0	0	0	1
0	0	0	0	0
0	0	0	0	0
0	1	0	0	0
0	0	0	0	0
0	0	0	0	0
0	0	0	0	0
0	0	0	0	0
0	0	0	0	0
0	0	0	0	0
0	0	0	0	0
0	0	0	0	0
0	0	0	0	0
0	0	0	0	0
0	0	0	0	0
0	0	0	0	0
0	0	0	0	0
0	0	0	0	0
0	0	0	0	0
0	0	0	0	0
0	0	0	0	0
0	0	0	0	0
0	0	0	0	0
0	0	0	0	0
0	0	0	0	0
0	0	0	0	0
0	0	0	0	0
0	0	0	0	0
0	0	0	0	0

0	0	0	0	0
0	0	0	0	0
0	0	0	0	0
0	0	0	0	0
0	0	0	0	0
0	0	0	0	0
0	0	0	0	0
0	0	0	0	0
0	0	0	0	0
0	0	0	0	0
0	0	0	0	0
0	0	0	0	0
0	0	0	0	0
0	0	0	0	0
0	0	0	0	0
0	0	0	0	0
0	0	0	0	0
0	0	0	0	0
0	0	0	0	0
0	0	0	0	0
0	0	0	0	0
0	0	0	0	0
0	0	0	0	0
0	0	0	0	0
0	0	0	0	0
0	0	0	0	0
0	0	0	0	0
0	0	0	0	0
0	0	0	0	0
0	0	0	0	0
0	0	0	0	0
0	0	0	0	0
0	0	0	0	0
0	0	0	0	0
0	0	0	0	0
0	0	0	0	0
0	0	0	0	0
0	0	0	0	0
0	0	0	0	0
0	0	0	0	0
0	0	0	0	0
0	0	0	0	0
0	0	0	0	0

0	0	0	0	0
0	0	0	0	0
0	0	0	0	0
0	0	0	0	0
0	0	0	0	0
0	0	0	0	0
0	0	0	0	0
0	0	0	0	0
0	0	0	0	0
0	0	0	0	0
0	0	0	0	0
0	0	0	0	0
0	0	0	0	0
0	0	0	0	0
0	0	0	0	0
0	0	0	0	0
0	0	0	0	0
0	0	0	0	0
0	0	0	0	0
0	0	0	0	0
0	0	0	0	0
0	0	0	0	0
0	0	1	0	0
0	0	1	0	0
0	0	1	0	0
0	0	1	0	0
0	0	1	0	0
0	0	1	0	0
0	0	0	0	0
0	0	0	0	0
0	0	0	0	0
0	0	1	0	0
0	1	0	1	0
0	0	0	0	0
0	1	0	0	1
0	1	1	0	0
0	0	0	0	0
0	0	0	1	1
0	0	0	0	0
0	0	0	0	1
0	0	0	0	0
0	0	0	0	0
0	0	0	0	0

0	0	0	0	0
0	0	0	0	0
0	0	0	0	0
0	0	0	0	0
0	0	0	0	0
0	0	0	0	0
0	0	0	0	0
0	0	0	0	0
0	0	0	0	0
0	0	0	0	0
0	0	0	0	0
0	0	0	0	0
0	0	0	0	0
0	0	0	0	0
0	0	0	0	0
0	0	0	0	0
0	0	0	0	0
0	0	0	0	0

**Larva (Quironimidae)**	**Muricidae**	**NEMATODA**	**Flabelligeridae**	**PHOXOCEPHALIDAE**	**DE**
0	0	0	0	0	
0	0	0	0	0	
0	0	0	0	0	
0	0	0	0	0	
0	0	0	0	0	
0	0	0	0	0	
0	0	0	0	0	
0	0	0	0	0	
0	0	0	0	0	
0	0	0	0	0	
0	0	0	0	0	
0	0	0	0	0	
0	0	0	0	0	
0	0	0	0	0	
0	0	0	0	0	
0	0	0	0	0	
0	0	0	0	0	
0	0	0	0	0	
0	0	0	0	0	
1	0	0	0	0	
0	0	0	0	0	
0	0	0	0	0	
0	0	0	0	0	

0	0	0	0	0
0	0	0	0	0
0	0	0	0	0
0	0	0	0	0
0	0	0	0	0
0	0	0	0	0
0	0	0	0	0
0	0	0	0	0
0	0	0	0	0
0	0	0	0	0
0	0	0	0	0
0	0	0	0	0
0	0	0	0	0
0	0	0	0	0
0	0	0	0	0
0	0	0	0	0
0	0	0	0	0
0	0	0	0	0
0	0	0	0	0
0	0	0	0	0
0	0	0	0	0
0	0	0	0	0
0	0	0	0	0
0	0	0	0	0
0	0	0	0	0
0	0	0	0	0
0	0	0	0	0
0	0	0	0	0
0	0	0	0	0
0	0	0	0	0
0	0	0	0	0
0	0	0	0	0
0	0	0	0	0
0	0	0	0	0
0	0	0	0	0
0	0	0	0	0
0	0	0	0	0
0	0	0	0	0
1	0	0	0	0
1	0	0	0	0
1	0	0	0	0
1	0	0	0	0
0	0	0	0	1

0	0	0	0	0
0	0	0	0	1
0	0	0	0	0
0	0	0	0	0
0	0	0	0	0
0	0	0	1	0
0	0	0	0	0
1	0	0	0	0
1	0	0	0	0
0	0	0	0	0
0	0	0	0	0
0	0	0	0	0
0	0	0	0	0
0	0	0	0	0
0	0	0	0	0
0	0	0	0	0
0	0	0	0	0
0	0	0	0	0
0	0	0	0	0
0	0	0	0	0
0	0	0	0	0
0	0	0	0	0
0	0	0	0	0
0	0	0	0	0
0	0	0	0	0
0	0	0	0	0
0	0	0	0	0
0	0	0	0	0
0	0	0	0	0
0	0	0	0	0
0	0	1	0	0
0	0	0	0	0
0	0	0	0	0
0	0	0	0	0
0	0	0	0	0
0	0	0	0	0
0	0	0	0	0
0	0	0	0	0
0	0	0	0	0
0	0	0	0	0
0	0	0	0	0
0	0	0	0	0
0	0	0	0	0

0	0	0	0	0
0	1	0	0	0
0	0	0	0	0
0	0	0	0	0
1	0	0	0	0
1	0	1	0	0
0	0	0	0	0
0	0	0	0	0
0	0	0	0	0
0	0	0	0	0
0	0	0	0	0
0	0	0	0	0
0	0	0	0	0
0	0	0	0	0
0	0	0	0	0
0	0	0	0	0
0	0	0	0	0
0	0	0	0	0
0	0	0	0	0
0	0	0	0	0
0	0	0	0	0
0	0	0	0	0
0	0	0	0	0
0	0	0	0	0
0	0	0	0	0
0	0	0	0	0

**Caridea**	**Upogebiidae**	**Sphaerodoridae**	**Luciferidae**	**Amphipoda**	**chaetognata**
0	0	0	1	0	0
0	0	0	0	0	0
0	0	0	0	0	0
0	0	0	0	0	0
0	0	1	0	1	0
0	0	0	0	1	0
0	0	0	0	0	0
0	0	0	0	0	0
0	0	0	0	0	0
0	0	0	0	0	0
0	0	0	0	0	0
0	0	0	0	0	0
0	0	0	0	0	0
1	0	0	0	0	0
0	1	0	0	0	0
0	0	0	0	0	0

0	0	0	0	0	0
0	0	0	0	0	0
0	0	0	0	0	0
0	0	0	0	0	0
0	0	0	0	0	0
0	0	0	0	0	0
0	0	0	0	0	0
0	0	0	0	0	0
0	0	0	0	0	0
0	0	0	0	0	0
0	0	0	0	0	0
0	0	0	0	0	0
0	0	0	0	0	0
0	0	0	0	0	0
0	0	0	0	0	0
0	0	0	0	0	0
0	0	0	0	0	0
0	0	0	0	0	0
0	0	0	0	0	0
0	0	0	0	0	0
0	0	0	0	0	0
0	0	0	0	0	0
0	0	0	0	0	0
0	0	0	0	0	0
0	0	0	0	0	0
0	0	0	0	0	0
0	0	0	0	0	0
0	0	0	0	0	0
0	0	0	0	0	0
0	0	0	0	0	0
0	0	0	0	0	0
0	0	0	0	0	0
0	0	0	0	0	0
0	0	0	0	0	0
0	0	0	0	0	0
0	0	0	0	0	0
0	0	0	0	0	0
0	0	0	0	0	0
0	0	0	0	0	0
0	0	0	0	1	1
0	0	0	0	1	1
0	0	0	0	1	1
0	0	0	0	0	1

0	0	0	0	0	1
0	0	0	0	0	1
0	0	0	0	0	0
0	0	0	0	1	0
0	0	0	0	1	0
0	0	0	0	0	0
0	0	0	0	0	0
0	0	0	0	1	0
0	0	0	0	1	0
0	0	0	0	0	0
0	0	0	0	0	0
0	0	0	0	0	0
0	0	0	0	0	0
0	0	0	0	0	0
0	0	0	0	0	0
0	0	0	0	0	0
0	0	0	0	1	0
0	0	0	0	0	0
0	0	0	0	0	0
0	0	0	0	0	0
0	0	0	0	0	0
0	0	0	0	0	0
0	0	0	0	0	0
0	0	0	0	0	0
0	0	0	0	0	0
0	0	0	0	0	0
0	0	0	0	1	0
0	0	0	0	0	0
0	0	0	0	1	0
0	0	0	0	0	0
0	0	0	0	0	0
0	0	0	0	0	0
0	0	0	0	0	0
0	0	0	0	0	0
0	0	0	0	0	0
0	0	0	0	0	0
0	0	0	0	0	0
0	0	0	0	0	0
0	0	0	1	0	0
0	1	0	0	0	0
0	0	0	0	0	1
0	0	0	0	0	0
0	0	0	0	0	0

0	0	0	0	0	0
0	0	0	0	0	1
0	0	0	0	0	0
0	0	0	0	0	0
0	0	0	0	0	0
0	0	0	0	0	0
0	0	0	0	0	0
0	0	0	0	0	0
0	0	0	0	0	0
0	0	0	0	0	0
0	0	0	0	0	0
0	0	0	0	0	0
0	0	0	0	0	0
0	0	0	0	0	0
0	0	0	0	0	0
0	0	0	0	0	0
0	0	0	0	0	0
0	0	0	0	0	0
0	0	0	0	0	0
0	0	0	0	0	0
0	0	0	0	0	0
0	0	0	0	0	0
0	0	0	0	0	0
0	0	0	0	0	0
0	0	0	0	0	0
0	0	0	0	0	0
0	0	0	0	0	0
0	0	0	0	0	0
0	0	0	0	0	0
0	0	0	0	0	0
0	0	0	0	0	0
0	0	0	0	0	0
0	0	0	0	0	0
**Scaphopoda**	**Acoetidae**	**Sigalionidae**	**Rissoidae**	**Mysidae**	**Hamineida**
0	0	0	0	0	0
0	0	0	0	0	0
0	0	0	0	0	0
0	0	0	0	0	0
0	0	0	0	0	0
0	0	0	0	0	0
0	0	0	0	0	0
0	0	0	0	0	0
0	0	0	0	0	0

0	0	0	0	0	0
0	0	0	0	0	0
0	0	0	0	0	0
0	0	0	0	0	0
0	0	0	0	0	0
0	0	0	0	0	0
0	0	0	0	0	0
0	0	0	0	0	0
0	0	0	0	0	0
0	0	0	0	0	0
0	0	0	0	0	0
0	0	0	0	0	0
0	0	0	0	0	0
0	0	0	0	0	0
0	0	0	0	0	0
0	0	0	0	0	0
0	0	0	0	0	0
0	0	0	0	0	0
0	0	0	0	0	0
0	0	0	0	0	0
0	0	0	0	0	0
0	0	0	0	0	0
0	0	0	0	0	0
0	0	0	0	0	0
0	0	0	0	0	0
0	0	0	0	0	0
0	0	0	0	0	0
0	0	0	0	0	0
0	0	0	0	0	0
0	0	0	0	0	0
0	0	0	0	0	0
0	0	0	0	0	0
0	0	0	0	0	0
0	0	0	0	0	0
0	0	0	0	0	0
0	0	0	0	0	0
0	0	0	0	0	0
0	0	0	0	0	0
0	0	0	0	0	0
0	0	0	0	0	0
0	0	0	0	0	0
0	0	0	0	0	0
0	0	0	0	0	0

0	0	0	0	0	0
0	0	0	0	0	0
0	0	0	0	0	0
0	0	0	1	1	0
0	0	0	0	0	0
0	0	0	0	0	0
0	0	0	0	0	0
0	0	0	0	0	0
0	0	0	0	0	0
0	0	0	0	0	0
0	0	0	0	0	0
0	0	0	0	0	0
0	0	0	1	0	0
0	0	0	0	0	0
0	0	0	0	0	0
0	0	0	0	0	0
0	0	0	0	0	0
0	0	0	0	0	0
0	0	0	0	0	0
0	0	0	0	0	0
0	0	0	0	0	0
0	0	0	0	0	0
0	0	0	0	0	0
0	0	0	0	0	0
0	0	0	0	0	0
0	0	0	0	0	0
0	0	0	0	0	0
0	0	0	0	0	0
0	0	0	0	0	0
0	0	0	0	0	0
0	0	0	0	0	0
0	0	0	0	0	0
0	0	0	0	0	0
0	0	0	0	0	0
0	0	0	0	0	0
0	0	0	0	0	0
0	0	0	0	0	0
0	0	0	0	0	0
0	0	0	0	0	0
0	0	0	0	0	0
0	0	0	0	0	0
0	0	0	0	0	0
0	0	0	0	0	0

0	0	0	0	0	0
1	0	1	0	1	0
0	0	0	0	1	0
0	0	0	0	0	0
0	1	1	0	0	1
0	0	0	0	0	0
0	0	0	1	0	0
0	0	0	0	0	0
0	0	0	0	1	0
0	0	0	0	1	0
0	0	0	0	0	0
0	0	0	0	0	0
0	0	0	0	0	0
0	0	0	0	0	0
0	0	0	0	0	0
0	0	0	0	0	0
0	0	0	0	0	0
0	0	0	0	0	0
0	0	0	0	0	0
0	0	0	0	0	0
0	0	0	0	0	0
0	0	0	0	0	0
0	0	0	0	0	0
0	0	0	0	0	0
0	0	0	0	0	0
0	0	0	0	0	0
0	0	0	0	0	0
0	0	0	0	0	0
0	0	0	0	0	0
0	0	0	0	0	0
0	0	0	0	0	0
0	0	0	0	0	0
0	0	0	0	0	0
0	0	0	0	0	0
0	0	0	0	0	0
0	0	0	0	0	0
0	0	0	0	0	0
0	0	0	0	0	0
0	0	0	0	0	0
0	0	0	0	0	0
**TROCHOCHAETIDAE**	**Naritidae**	**Gobiidae**	**Hyalidae**	**Aristidae**	**Apseudellidae**
0	0	0	0	0	0
0	0	0	0	0	0

0	0	0	0	0	0
0	0	0	0	0	0
0	0	0	0	0	0
0	0	0	0	0	0
0	0	0	0	0	0
0	0	0	0	0	0
0	0	0	0	0	0
0	0	0	0	0	0
0	0	0	0	0	0
0	0	0	0	0	0
0	0	0	0	0	0
0	0	0	0	0	0
0	0	0	0	0	0
0	0	0	0	0	0
0	0	0	0	0	0
0	0	0	0	0	0
0	0	0	0	0	0
0	0	0	0	0	0
0	0	0	0	0	0
0	0	0	0	0	0
0	0	0	0	0	0
0	0	0	0	0	0
0	0	0	0	0	0
0	0	0	0	0	0
0	0	0	0	0	0
0	0	0	0	0	0
0	0	0	0	0	0
0	0	0	0	0	0
0	0	0	0	0	0
0	0	0	0	0	0
0	0	0	0	0	0
0	0	0	0	0	0
0	0	0	0	0	0
0	0	0	0	0	0
0	0	0	0	0	0
0	0	0	0	0	0
0	0	0	0	0	0
0	0	0	0	0	0
0	0	0	0	0	0
0	0	0	0	0	0
0	0	0	0	0	0
0	0	0	0	0	0
0	0	0	0	0	0

0	0	0	0	0	0
0	0	0	0	0	0
0	0	0	0	0	0
0	0	0	0	0	0
0	0	0	0	0	0
0	0	0	0	0	0
0	0	0	0	0	0
0	0	0	0	0	0
0	0	0	0	0	0
0	0	0	0	0	0
0	0	0	0	0	0
0	0	0	0	0	0
0	0	0	0	0	0
0	0	0	0	0	0
0	0	0	0	0	0
0	0	0	0	0	0
0	0	0	0	0	0
0	0	0	0	0	0
0	0	0	0	0	0
0	0	0	0	0	0
0	0	0	0	0	0
0	0	0	0	0	0
0	0	0	0	0	0
0	0	0	0	0	0
0	0	0	0	0	0
0	0	0	0	0	0
0	0	0	0	0	0
0	0	0	0	0	0
0	0	0	0	0	0
0	0	0	0	0	0
0	0	0	0	0	0
0	0	0	0	0	0
0	0	0	0	0	0
0	0	0	0	0	0
0	0	0	0	0	0
0	0	0	0	0	0
0	0	0	0	0	0
0	0	0	0	0	0
0	0	0	0	0	0
0	0	0	0	0	0
0	0	0	0	0	0
0	0	0	0	0	0
0	0	0	0	0	0

0	0	0	0	0	0
0	0	0	0	0	0
0	0	0	0	0	0
0	0	0	0	0	0
0	0	0	0	0	0
0	0	0	0	0	0
0	0	0	0	0	0
0	0	0	0	0	0
0	0	0	0	0	0
0	0	0	0	0	0
1	0	0	0	0	0
0	0	0	1	0	1
0	0	0	0	0	0
0	0	0	0	0	0
0	1	0	0	0	0
0	0	0	1	1	0
0	0	0	0	0	0
0	0	0	0	0	0
0	0	1	0	0	0
0	0	0	0	0	0
0	0	0	0	0	0
0	0	0	0	0	0
0	0	0	0	0	0
0	0	0	0	0	0
0	0	0	0	0	0
0	0	0	0	0	0
0	0	0	0	0	0
0	0	0	0	0	0
0	0	0	0	0	0
0	0	0	0	0	0
0	0	0	0	0	0
0	0	0	0	0	0
0	0	0	0	0	0
0	0	0	0	0	0
0	0	0	0	0	0
0	0	0	0	0	0
0	0	0	0	0	0
0	0	0	0	0	0
0	0	0	0	0	0
0	0	0	0	0	0
0	0	0	0	0	0
0	0	0	0	0	0
0	0	0	0	0	0

0	0	0	0	0	0
0	0	0	0	0	0
0	0	0	0	0	0
0	0	0	0	0	0
**PLATYISCHNOPIDAE**	**Trapezidae**	**LARVA DE THALASSINIDAE**	**Tanallidae**	**Amphitoidea**	**LEPTOCHAELLIDAE**
0	0	0	0	0	0
0	0	0	0	0	0
0	0	0	0	0	0
0	0	0	0	0	0
0	0	0	0	0	0
0	0	0	0	0	0
0	0	0	0	0	0
0	0	0	0	0	0
0	0	0	0	0	0
0	0	0	0	0	0
0	0	0	0	0	0
0	0	0	0	0	0
0	0	0	0	0	0
0	0	0	0	0	0
0	0	0	0	0	0
0	0	0	0	0	0
0	0	0	0	0	0
0	0	0	0	0	0
0	0	0	0	0	0
0	0	0	0	0	0
0	0	0	0	0	0
0	0	0	0	0	0
0	0	0	0	0	0
0	0	0	0	0	0
0	0	0	0	0	0
0	0	0	0	0	0
0	0	0	0	0	0
0	0	0	0	0	0
0	0	0	0	0	0
0	0	0	0	0	0
0	0	0	0	0	0
0	0	0	0	0	0
0	0	0	0	0	0
0	0	0	0	0	0
0	0	0	0	0	0
0	0	0	0	0	0
0	0	0	0	0	0

0	0	0	0	0	0
0	0	0	0	0	0
0	0	0	0	0	0
0	0	0	0	0	0
0	0	0	0	0	0
0	0	0	0	0	0
0	0	0	0	0	0
0	0	0	0	0	0
0	0	0	0	0	0
0	0	0	0	0	0
0	0	0	0	0	0
0	0	0	0	0	0
0	0	0	0	0	0
0	0	0	0	0	0
0	0	0	0	0	0
0	0	0	0	0	0
0	0	0	0	0	0
0	0	0	0	0	0
0	0	0	0	0	0
0	0	0	0	0	0
0	0	0	0	0	0
0	0	0	0	0	0
0	0	0	0	0	0
0	0	0	0	0	0
0	0	0	0	0	0
0	0	0	0	0	0
0	0	0	0	0	0
0	0	0	0	0	0
0	0	0	0	0	0
0	0	0	0	0	0
0	0	0	0	0	0
0	0	0	0	0	0
0	0	0	0	0	0
0	0	0	0	0	0
0	0	0	0	0	0
0	0	0	0	0	0
0	0	0	0	0	0
0	0	0	0	0	0
0	0	0	0	0	0
0	0	0	0	0	0
0	0	0	0	0	0
0	0	0	0	0	0
0	0	0	0	0	0

0	0	0	0	0	0
0	0	0	0	0	0
0	0	0	0	0	0
0	0	0	0	0	0
0	0	0	0	0	0
0	0	0	0	0	0
0	0	0	0	0	0
0	0	0	0	0	0
0	0	0	0	0	0
0	0	0	0	0	0
0	0	0	0	0	0
0	0	0	0	0	0
0	0	0	0	0	0
0	0	0	0	0	0
0	0	0	0	0	0
0	0	0	0	0	0
0	0	0	0	0	0
0	0	0	0	0	0
0	0	0	0	0	0
1	0	0	0	0	0
0	1	1	1	1	1
0	0	0	0	0	0
0	0	0	0	0	0
0	0	0	0	0	0
0	0	0	0	0	0
0	0	0	0	0	0
0	0	0	0	0	0
0	0	0	0	0	0
0	0	0	0	0	0
0	0	0	0	0	0
0	0	0	0	0	0
0	0	0	0	0	0
0	0	0	0	0	0
0	0	0	0	0	0
0	0	0	0	0	0
0	0	0	0	0	0
0	0	0	0	0	0
0	0	0	0	0	0
0	0	0	0	0	0
0	0	0	0	0	0
0	0	0	0	0	0
0	0	0	0	0	0
0	0	0	0	0	0

0	0	0	0	0	0
0	0	0	0	0	0
0	0	0	0	0	0
0	0	0	0	0	0
0	0	0	0	0	0
0	0	0	0	0	0
0	0	0	0	0	0
0	0	0	0	0	0
0	0	0	0	0	0
0	0	0	0	0	0
0	0	0	0	0	0
0	0	0	0	0	0

CYPROIDEIDEA	Phasianellidae	Portunidae
0	0	0
0	0	0
0	0	0
0	0	0
0	0	0
0	0	0
0	0	0
0	0	0
0	0	0
0	0	0
0	0	0
0	0	0
0	0	0
0	0	0
0	0	0
0	0	0
0	0	0
0	0	0
0	0	0
0	0	0
0	0	0
0	0	0
0	0	0
0	0	0
0	0	0
0	0	0
0	0	0
0	0	0
0	0	0
0	0	0

0	0	0
0	0	0
0	0	0
0	0	0
0	0	0
0	0	0
0	0	0
0	0	0
0	0	0
0	0	0
0	0	0
0	0	0
0	0	0
0	0	0
0	0	0
0	0	0
0	0	0
0	0	0
0	0	0
0	0	0
0	0	0
0	0	0
0	0	0
0	0	0
0	0	0
0	0	0
0	0	0
0	0	0
0	0	0
0	0	0
0	0	0
0	0	0
0	0	0
0	0	0
0	1	0
0	0	0
0	0	0
0	0	0
0	0	0
0	0	0
0	0	0
0	0	0
0	0	0

0	0	0
0	0	0
0	0	0
0	0	0
0	0	0
0	0	0
0	0	0
0	0	0
0	0	0
0	0	0
0	0	0
0	0	0
0	0	0
0	0	0
0	0	0
0	0	0
0	0	0
0	0	0
0	0	0
0	0	0
0	0	0
0	0	0
0	0	0
0	0	0
0	0	0
0	0	0
0	0	0
1	0	0
0	0	0
0	0	0
0	0	0
0	0	0
0	0	0
0	0	0
0	0	0
0	0	0
0	0	0
0	0	0
0	0	0
0	0	0
0	0	0
0	0	0
0	0	0

0	0	0
0	0	0
0	0	0
0	0	0
0	0	0
0	0	0
0	0	0
0	0	0
0	0	0
0	0	0
0	0	0
0	0	0
0	0	0
0	0	0
0	0	0
0	0	0
0	0	0
0	0	0
0	0	0
