# Supplementary material for: Drivers of benthic metacommunity structure along tropical estuaries
Source: Sci Rep. 2020 Feb 3;10:1739. doi: 10.1038/s41598-020-58631-1 (PMC6997391; doi:10.1038/s41598-020-58631-1)
Supplement: Supplementary file 3 — Supplementary Dataset - Environmental Data. [file 41598_2020_58631_MOESM3_ESM.pdf]

Drivers of benthic metacommunity structure along tropical estuaries

Andreia Teixeira Alves[1*], Danielle Katharine Petsch[2], Francisco Barros[1]

*Corresponding author e-mail address: dea_alves106@yahoo.com.br ; Telephone: +55 71991542200 1.

1. Laboratório de Ecologia Bentônica (LEB), Instituto de Biologia, Programa de Pós Graduação em Ecologia e Biomonitoramento, Universidade Federal da Bahia, Brazil

2. Núcleo de Pesquisas em Limnologia, Ictiologia e Aquicultura (Nupelia), Programa de Pós- Graduação em Ecologia de Ambientes Aquáticos Continentais (PEA), Universidade Estadual de Maringá, Brazil

Supplementary Dataset Environmental Data

Estuary	Month	Year	Sites	Salinity	Pebble	Gravel
Subaé	March	2013	#1	35,1	0	0,45
Subaé	March	2013	#2	35,07	0	0,15
Subaé	March	2013	#3	35,32	0	0,44
Subaé	March	2013	#4	34,1	0	0
Subaé	March	2013	#5	32,56	0	4,67
Subaé	March	2013	#6	28,68	0	0,46
Subaé	March	2013	#7	24,8	0	3,52
Subaé	March	2013	#8	17,16	0	0,67
Subaé	March	2013	#9	13,3	0	0
Subaé	March	2013	#10	10,89	0	0,28
Subaé	March	2013	#11	10,17	0	4,15
Subaé	April	2011	#1	35	0	0
Subaé	April	2011	#2	36	0	0,22
Subaé	April	2011	#3	36	0	0
Subaé	April	2011	#4	35	0	0,02
Subaé	April	2011	#5	32	0	4,07
Subaé	April	2011	#6	26	0,63	2,38
Subaé	April	2011	#7	24	0,22	1,76
Subaé	April	2011	#8	15	0,22	1,29
Subaé	April	2011	#9	11	0,2	0,47
Subaé	April	2011	#10	15	0	0,43
Subaé	April	2011	#11	11	0	0,49
Subaé	December	2009	#1	40	0,08	1,78
Subaé	December	2009	#2	40	12,2	55,18
Subaé	December	2009	#3	41,5	0	0,19
Subaé	December	2009	#4	40,5	0	0,14
Subaé	December	2009	#5	40	2,78	2,94
Subaé	December	2009	#6	37,5	0	0,01
Subaé	December	2009	#7	32	0,53	0,13

Subaé	December	2009	#8	23	0,22	1,29
Subaé	December	2009	#9	21	0	0,01
Subaé	December	2009	#10	16	0,17	1,05
Subaé	December	2009	#11	25	0,63	5,57
Subaé	March	2006	#1	37,88	0	0
Subaé	March	2006	#2	38,12	22,9	26,91
Subaé	March	2006	#3	39,35	0	0
Subaé	March	2006	#4	36,98	0	0
Subaé	March	2006	#5	37	0	0
Subaé	March	2006	#6	35,28	0	0
Subaé	March	2006	#7	33,45	5,24	5,4
Subaé	March	2006	#8	25,65	5,45	6,98
Subaé	March	2006	#9	19,34	3,19	4
Subaé	March	2006	#10	12,08	9,51	9,47
Subaé	March	2006	#11	4,5	9,85	9,87
Subaé	June	2004	#1	27,52	0	0,02
Subaé	June	2004	#2	26,14	21,08	31,86
Subaé	June	2004	#3	24,44	0	0,04
Subaé	June	2004	#4	23,9	0	0
Subaé	June	2004	#5	21,25	0,77	0,38
Subaé	June	2004	#6	15,94	0	0,01
Subaé	June	2004	#7	9,75	0,2	1,19
Subaé	June	2004	#8	4,98	0	0,3
Subaé	June	2004	#9	1,87	0	0,32
Subaé	June	2004	#10	0,49	0,4	2,83
Subaé	June	2004	#11	0,17	0	0,96
Jaguaripe	August	2014	#1	40,9	0,1	0,38
Jaguaripe	August	2014	#2	34,2	3,27	3,75
Jaguaripe	August	2014	#3	28,6	0	0,15
Jaguaripe	August	2014	#4	27,3	0	0,17
Jaguaripe	August	2014	#5	28,7	0	0
Jaguaripe	August	2014	#6	19,9	0	0
Jaguaripe	August	2014	#7	14,4	0	2,61
Jaguaripe	August	2014	#8	1,5	0	0,88
Jaguaripe	August	2014	#9	0,24	6,65	18,04
Jaguaripe	August	2014	#10	0,1	0	0,71
Jaguaripe	August	2010	#1	28,02	0,07	0,23
Jaguaripe	August	2010	#2	23,13	1,29	1,81
Jaguaripe	August	2010	#3	18,58	0,06	0,15
Jaguaripe	August	2010	#4	15,91	0,02	0,07
Jaguaripe	August	2010	#5	15,36	0,02	0,02
Jaguaripe	August	2010	#6	10,38	0,01	0,17
Jaguaripe	August	2010	#7	7,04	3,54	1,99

Jaguaripe	August	2010	#8	1,7	0,14	0,46
Jaguaripe	August	2010	#9	0,24	4,19	8,44
Jaguaripe	August	2010	#10	1,11	2,01	2,47
Jaguaripe	August	2007	#1	16,7	0,06	0,25
Jaguaripe	August	2007	#2	10,9	0,04	0,5
Jaguaripe	August	2007	#3	4,62	0,06	0,12
Jaguaripe	August	2007	#4	1,13	0	0
Jaguaripe	August	2007	#5	0,53	0,05	0,02
Jaguaripe	August	2007	#6	0,23	0,03	0,36
Jaguaripe	August	2007	#7	0,14	0,79	2,21
Jaguaripe	August	2007	#8	0,12	0,41	0,46
Jaguaripe	August	2007	#9	0,12	0,64	0,95
Jaguaripe	August	2007	#10	0,11	0,12	0,3
Jaguaripe	May	2006	#1	26,45	0,06	0,05
Jaguaripe	May	2006	#2	24,29	0,55	1,19
Jaguaripe	May	2006	#3	22,53	0,13	0,17
Jaguaripe	May	2006	#4	19,29	0,06	0,03
Jaguaripe	May	2006	#5	16,84	0	0,05
Jaguaripe	May	2006	#6	11,01	0	0,14
Jaguaripe	May	2006	#7	6,57	9,82	1,16
Jaguaripe	May	2006	#8	3,48	0	0,04
Jaguaripe	May	2006	#9	0,36	5,28	6,34
Jaguaripe	May	2006	#10	0,11	5,9	6,41
Paraguaçu	August	2014	#1	32,77	0	0,32
Paraguaçu	August	2014	#2	38,7	0	0,46
Paraguaçu	August	2014	#3	30,71	0	0
Paraguaçu	August	2014	#4	37	0	0,73
Paraguaçu	August	2014	#5	20,85	0	0,64
Paraguaçu	August	2014	#6	35,7	0	3,36
Paraguaçu	August	2014	#7	12,56	2,02	4,31
Paraguaçu	August	2014	#8	26,8	0	0,73
Paraguaçu	August	2014	#9	6,68	0	1,95
Paraguaçu	August	2014	#10	21,3	0	6,22
Paraguaçu	June	2011	#1	34	12,02	5,73
Paraguaçu	June	2011	#2	33	1,74	0,71
Paraguaçu	June	2011	#3	31	9,8	4,79
Paraguaçu	June	2011	#4	26	2,84	5,32
Paraguaçu	June	2011	#5	20	0,24	1,98
Paraguaçu	June	2011	#6	27	0,29	1,69
Paraguaçu	June	2011	#7	15	0,34	0,86
Paraguaçu	June	2011	#8	11	0	0,22
Paraguaçu	June	2011	#9	9	0,26	1,49
Paraguaçu	June	2011	#10	3	0,13	3,25

Paraguaçu	May	2005	#1	30,08	12,18	7,16
Paraguaçu	May	2005	#2	29,14	0,67	0,94
Paraguaçu	May	2005	#3	28,3	0,02	0,23
Paraguaçu	May	2005	#4	26,7	0	0,19
Paraguaçu	May	2005	#5	21,09	5,26	7,81
Paraguaçu	May	2005	#6	12,92	0,65	6,5
Paraguaçu	May	2005	#7	7,72	0	0,94
Paraguaçu	May	2005	#8	4,53	1,58	8,77
Paraguaçu	May	2005	#9	2,2	0	0,25
Paraguaçu	May	2005	#10	0,6	0	0,63
Paraguaçu	December	2005	#1	34,22	0,62	0,45
Paraguaçu	December	2005	#2	33,39	1,33	0,52
Paraguaçu	December	2005	#3	32,84	0,04	0,11
Paraguaçu	December	2005	#4	31,66	0,12	0
Paraguaçu	December	2005	#5	28,23	3,71	7,21
Paraguaçu	December	2005	#6	21,45	0,3	3,7
Paraguaçu	December	2005	#7	14,96	0,39	0,5
Paraguaçu	December	2005	#8	12,13	1,23	5,12
Paraguaçu	December	2005	#9	8,94	0	0,13
Paraguaçu	December	2005	#10	7,78	0,15	0,81

**Very coarse sand**	**Coarse sand**	**Medium sand**	**Fine sand**	**Very fine sand**	**Silt and clay**
0,12	0,65	1,28	4,51	21,09	71,9
0,46	0,45	1,85	2,28	5,59	89,24
0,17	0,26	3,21	5,6	5,72	84,61
0,57	1,15	2,43	4,93	14,02	76,9
5,27	8,68	6,68	17,88	8,43	48,38
0,57	1,2	2,25	60,62	21,66	13,24
20,88	53,91	21,42	0,27	0	0
4,83	36,46	54,26	3,68	0	0,1
1,13	24,7	65,08	8,26	0,13	0,71
0,49	1,39	3,29	25,52	22,68	46,34
13,97	23,09	8,95	3,26	3,49	43,09
0,19	3,47	10,23	15,72	60,5	9,88
1,8	8,61	13,07	24,68	43,18	8,45
1,97	8,17	14,55	33,56	35,62	6,13
0,2	0,75	2,72	48,74	42,28	5,29
5,87	15,12	26,32	30,18	10,13	8,32
9,17	45,1	37,87	4,52	0,28	0,05
7,28	12,82	48,53	28,77	0,47	0,15
10,68	37,01	45,79	4,85	0,1	0,06

3,3	21,54	62,5	11,68	0,2	0,11
6,01	34,2	52,89	5,72	0,48	0,28
5,44	38,73	46,14	8,99	0,17	0,04
6,81	16,4	14,9	19,8	28,5	11,73
21,44	3,97	1,91	1,69	3,06	0,55
0,18	9,31	23,78	20,52	25,34	20,68
0,64	3,21	9,27	38,29	20,33	28,12
5,73	9,88	18,65	49,07	9,61	1,34
0,01	1,24	27,82	68,34	2,37	0,2
0,92	11,17	35,02	46,52	5,38	0,33
10,68	37,01	45,79	4,85	0,1	0,06
0,28	13,28	73,94	12,17	0,2	0,11
11,03	45,52	35,56	6,26	0,25	0,15
21,14	46,35	26,14	0,16	0	0
0	0,02	0,22	0,39	23,81	75,56
10,55	10,41	7,34	4,11	4,82	12,97
0	0,02	0,09	0,26	2,12	97,51
12,07	12,09	0,18	8,21	11,8	55,65
0	18,11	0,87	4,17	5,85	70,99
0	5,65	0,59	64,88	9,15	19,73
13,44	25,88	40,26	9,23	0,38	0,17
20,38	46,92	17,85	1,14	0,08	1,2
13,69	60,23	16,98	0,5	0,14	1,26
18,93	9,71	0,51	5,94	15,06	30,87
21,47	31,08	17,33	4,57	2,01	3,81
0	0,36	0,72	2,47	22,63	73,8
11,18	6,07	3,35	4,63	7,39	14,45
0,18	0,16	0,04	0,29	0,98	98,3
0,67	0,55	0,49	4,47	2,14	91,67
1,41	1,08	6,25	58,57	16,95	14,59
0,03	0,54	51,85	44,98	1,24	1,35
4,9	24,92	50,82	16,34	0,08	1,54
1,54	19,7	51,35	25,37	0,62	1,13
1,66	23,67	66,6	5,69	0,32	1,74
16,82	42,07	29,73	2,55	0,67	4,93
5,55	42,62	44,86	5,46	0,1	0,45
1,91	4,17	40,47	28,89	15,48	8,59
4,99	8,4	34	37,03	3,92	4,64
0,53	5,24	21,54	54,31	12,81	5,42
1,55	4,64	26,64	54,66	6,32	6,03
0,04	0,36	13,9	83,32	1,65	0,74
0,39	15,41	70,63	13,36	0,11	0,1
6,46	24,01	33,24	27,47	4,21	2

1,35	12,22	26,46	48	7,88	3,21
29,66	25,55	15,6	2,65	0,35	1,51
3,29	58,88	32	3,61	0,36	1,15
1,16	4,49	57,04	25,63	8,35	3,02
2,73	6,12	37,34	43,56	4,81	2,34
0,33	4,61	16,37	57,19	16,57	4,71
0,63	2,12	11,17	74,44	8,15	3,4
0,06	0,66	13,97	79,12	5,06	1,09
1,16	11,48	51,44	32,44	2,45	0,84
6,41	16,72	30,83	34,75	3,5	0,92
1,46	12,45	28,42	45,97	8,89	2,21
16,49	38,85	31,38	8,55	4,28	0,78
6,47	42,77	32,71	8,05	4,54	0,96
1,18	5,8	65,82	23,2	3,64	0,06
1,44	4,6	38,58	45,52	7,43	1,9
0,27	7,95	18,64	56,67	15,16	1,12
0,01	0,99	4,01	89,04	5,88	0,06
0,02	1,17	14,34	80,73	3,64	0,04
1,84	9,85	34,32	50,84	2,71	0,06
6,4	15,22	30,78	40,78	3,58	0,24
2,49	16,67	31,18	40,76	7	1,03
4,01	27,14	54,4	10,47	1,98	0,42
2,06	40,38	43,44	9,43	2,93	1,35
0,38	3,51	64,83	24,82	5,94	0,4
1,76	5,36	39,45	48,13	3,07	0,49
0,2	0,63	8,93	60,6	21,75	7,59
0,32	0,72	2,86	79,63	12,26	4,12
0,11	0,46	13,67	73,32	9,9	2,48
1,27	9,19	49,38	33,13	4,52	2,37
6,36	14,94	28,47	35,99	2,72	0,53
0,53	8,45	27,64	49,16	11,79	2,39
15,8	25,01	24,13	12,52	10,51	0,41
14,08	29,05	22,7	11,11	10,34	0,4
1,13	2,96	5,54	12,91	5,49	71,66
1,1	2,05	18,72	53,7	3,92	20,04
0	0,02	26,86	72,26	0,55	0,3
2,85	4,99	6,74	3,61	1,88	79,21
1,81	26	12,54	25,8	12,93	20,29
9,45	31,17	25,98	13,04	2,1	14,9
18,8	40,61	17,27	6,13	0,59	10,26
7,32	63,99	27,22	0,68	0	0,08
13,02	53,75	29,55	1,59	0	0,13
32,56	47,48	11,08	2,53	0,07	0,08

10,08	21	39,78	4,21	0,77	6,41
1,01	5,21	20,34	55,49	4,1	11,41
3,56	5,79	21,06	43,21	2,25	9,55
16,79	31,67	24,75	2,65	0,13	15,86
6,47	18,2	18,55	36,03	8,88	9,65
4,58	12,81	23,3	40,84	5,4	11,09
5,19	35,06	35,73	9,19	1,32	12,3
6,23	60,36	31,91	1,27	0	0
11,63	43,51	39,16	3,83	0,06	0,07
19,97	50,5	24,57	1,34	0	0,24
3,84	9,99	17,48	4,77	0,98	43,61
0,65	6,25	13,4	40,99	6,84	30,26
0,54	3,41	43,65	29,27	1,08	21,81
0,3	0,84	1,89	3,65	1,16	91,98
4,74	14,34	25,78	28,24	1,53	12,31
12,81	28,81	16,65	22,17	3,33	9,09
3,41	39,8	34,87	11,55	1,82	7,62
20,22	49,59	17,35	2,02	0,07	0,39
3,43	50,97	40,55	4,54	0,12	0,15
6,08	43,41	43,93	5,32	0,24	0,38
0,66	1,73	4,68	1,03	0,24	90,58
0,65	6,42	17,15	42,26	5	26,66
0,46	2,88	48,23	24,63	1,14	22,51
0,33	0,82	2,18	3,63	1,24	91,69
5,32	12,18	27,93	30,41	1,4	11,84
14,12	26,14	20,24	22,32	3,38	9,79
3,77	34,46	40,27	10,62	1,9	8,1
23,31	45,88	22,1	1,82	0,1	0,44
4,9	44,56	44,24	3,69	0,13	2,35
8,1	39,7	45,79	4,62	0,3	0,51
