# Supplementary material for: Drivers of benthic metacommunity structure along tropical estuaries
Source: Sci Rep. 2020 Feb 3;10:1739. doi: 10.1038/s41598-020-58631-1 (PMC6997391; doi:10.1038/s41598-020-58631-1)
Supplement: Supplementary file 4 — Supplementary Dataset - Spatial Coordinates. [file 41598_2020_58631_MOESM4_ESM.pdf]

Drivers of benthic metacommunity structure along tropical estuaries

Andreia Teixeira Alves[1*], Danielle Katharine Petsch[2], Francisco Barros[1]

*Corresponding author e-mail address: dea_alves106@yahoo.com.br ; Telephone: +55 71991542200 1.

1. Laboratório de Ecologia Bentônica (LEB), Instituto de Biologia, Programa de Pós Graduação em Ecologia e Biomonitoramento, Universidade Federal da Bahia, Brazil

2. Núcleo de Pesquisas em Limnologia, Ictiologia e Aquicultura (Nupelia), Programa de Pós- Graduação em Ecologia de Ambientes Aquáticos Continentais (PEA), Universidade Estadual de Maringá, Brazil

Supplementary Dataset Spatial Coordinates

Estuary	Sites	Latitude	Longitude
Subaé	#1	12°40'4.62°S	38°39'39.67°O
Subaé	#2	12°39'0.03°S	38°39'39.72°O
Subaé	#3	12°38'27.76°S	38°39'56.40°O
Subaé	#4	12°38'11.66°S	38°40'29.51°O
Subaé	#5	12°37'55.65°S	38°41'2.68°O
Subaé	#6	12°37'24.70°S	38°42'5.76°O
Subaé	#7	12°36'14.44°S	38°42'3.16°O
Subaé	#8	12°35'19.30°S	38°41'43.84°O
Subaé	#9	12°34'18.48°S	38°41'31.35°O
Subaé	#10	12°33'50.58°S	38°41'31.31°O
Subaé	#11	12°33'55.86°S	38°41'39.03°O
Jaguaripe	#1	13°7'13.67°S	38°50'23.38°O
Jaguaripe	#2	13°6'45.91°S	38°51'38.09°O
Jaguaripe	#3	13°6'31.61°S	38°53'14.94°O
Jaguaripe	#4	13°6'31.61°S	38°54'55.88°O
Jaguaripe	#5	13°6'5.26°S	38°56'6.82°O
Jaguaripe	#6	13°5'21.37°S	38°57'24.88°O
Jaguaripe	#7	13°4'4.34°S	38°57'31.30°O
Jaguaripe	#8	13°2'59.46°S	38°57'13.08°O
Jaguaripe	#9	13°2'3.64°S	38°58'47.82°O
Jaguaripe	#10	13°2'1.99°S	38°59'49.34°O
Paraguaçu	#1	12°50'3.32°S	38°47'22.40°O
Paraguaçu	#2	12°51'3.59°S	38°49'59.61°O
Paraguaçu	#3	12°49'22.01°S	38°51'40.00°O
Paraguaçu	#4	12°47'24.99°S	38°51'50.01°O
Paraguaçu	#5	12°45'30.61°S	38°52'26.39°O
Paraguaçu	#6	12°44'8.88°S	38°53'3.47°O
Paraguaçu	#7	12°43'46.58°S	38°54'11.53°O
Paraguaçu	#8	12°43'48.70°S	38°54'47.15°O

Paraguaçu	#9	12°43'52.69°S	38°55'19.91°O
Paraguaçu	#10	12°42'38.51°S	38°56'8.15°O
